# Ab Initio Study of Novel Phase‐Change Heterostructures

**DOI:** 10.1002/advs.202402375

**Published:** 2024-05-29

**Authors:** Riccardo Piombo, Simone Ritarossi, Riccardo Mazzarello

**Affiliations:** ^1^ Dipartimento di Fisica Università di Roma “La Sapienza” 00185 Rome Italy

**Keywords:** chalcogenide phase‐change materials, neuromorphic computing, phase‐change heterostructures

## Abstract

Neuromorphic devices constitute a novel approach to computing that takes inspiration from the brain to unify the processing and storage units. Memories based on phase‐change materials (PCMs) are potential candidates for such devices due to their non‐volatility and excellent scalability, however their use is hindered by their conductance variability and temporal drift in resistance. Recently, it has been shown that the utilization of phase‐change heterostructures consisting of nanolayers of the Sb_2_Te_3_ PCM interleaved with a transition‐metal dichalcogenide, acting as a confinement material, strongly mitigates these problems. In this work, superlattice heterostructures made of TiTe_2_ and two prototypical PCMs, respectively GeTe and Ge_2_Sb_2_Te_5_ are considered. By performing ab initio molecular dynamics simulations, it is shown that it is possible to switch the PCMs without destroying the superlattice structure and without diffusion of the atoms of the PCM across the TiTe_2_ nanolayers. In particular, the model containing Ge_2_Sb_2_Te_5_ shows weak coupling between the two materials during the switching process, which, combined with the high stability of the amorphous state of Ge_2_Sb_2_Te_5_, makes it a very promising candidate for neuromorphic computing applications.

## Graphical abstract

Superlattice heterostructures consisting of alternating ultrathin layers of TiTe_2_ and prototypical phase‐change materials (PCMs), namely GeTe and Ge_2_Sb_2_Te_5_, are investigated by ab initio molecular dynamics. It is shown that the PCM layers can switch without structural disruption or atomic diffusion across TiTe_2_ nanolayers. The Ge_2_Sb_2_Te_5_ model, particularly, exhibits weak coupling during switching, along with high stability in its amorphous state, making it a strong candidate for neuromorphic applications.

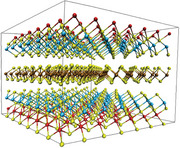

## 1 Introduction

The current information technology era is witnessing an unprecedented increase in the global demand for data storage and processing, driven by data‐centric computation, artificial intelligence, and mobile electronics. It has become apparent that traditional computing devices, which are based on the von Neumann architecture, cannot cope with this demand. This architecture creates a performance bottleneck due to the physical separation of the processing and memory units, which requires a constant back‐and‐forth data transfer between these units. Thus, there is a pressing need to develop new computing architectures beyond the von Neumann paradigms.

Neuromorphic computing is an auspicious approach that emulates the behavior of the brain to achieve the unification of computing and storage,^[^ ^1^ ^]^ which would enable to overcome the von Neumann bottleneck. Neuromorphic computing encompasses several concepts and technologies such as in‐memory computing, deep neural networks (NNs), and spiking NNs.^[^ ^2^, ^3^, ^4^ ^]^ Although a great deal of progress has been made in implementing these concepts using standard CMOS technology,^[^ ^4^, ^5^, ^6^ ^]^ resistance‐based memories, such as phase‐change memories,^[^ ^7^, ^8^ ^]^ offer key advantages such as non‐volatility and potentially superior scalability.^[^ ^4^ ^]^

Phase‐change memories exploit the ability of phase‐change materials (PCMs) to switch rapidly and reversibly between a crystalline and an amorphous state, exhibiting pronounced resistivity contrast. The transitions are induced by applying proper electrical pulses to the memory cells to increase their temperature via the Joule effect. Furthermore, partly amorphous, partly crystalline intermediate states can be obtained by tuning the height and width of the pulses. The combination of these properties and the non‐volatility makes PCMs suitable for in‐memory computing applications^[^ ^4^ ^]^ and for emulating integrate‐and‐fire^[^ ^9^ ^]^ and synaptic behavior.^[^ ^10^ ^]^

However, the intercell and intracell conductance variability and the resistance drift of the amorphous state are serious drawbacks. The cell variability originates from the atomic migration induced by the electrical pulses and the statistical variability of crystallization.^[^ ^11^, ^12^ ^]^ In contrast, the temporal drift of the resistance is due to the structural relaxation (aging) of the amorphous phase. These drawbacks can be partly cured by a clever device setup.^[^ ^13^, ^14^ ^]^

Recently, Ding et al. proposed a novel type of phase‐change cell to surmount these problems.^[^ ^15^ ^]^ This cell contains a phase‐change heterostructure (PCH), which consists of alternately grown nanolayers of a switchable PCM and a confinement material (CM). They chose Sb_2_Te_3_ as PCM and the transition‐metal dichalcogenide TiTe_2_ as CM. Due to the higher melting point of TiTe_2_ with respect to Sb_2_Te_3_, the CM layers remain crystalline during cycling and prevent the atomic migration along the pulsing direction, whereas the nanoconfinement of the PCM suppresses structural relaxation. As a result, cell variability and drift are significantly reduced, as compared to cells based on standard PCMs, enabling the implementation of robust and accurate iterative RESET and cumulative SET operations. Furthermore, the cycling endurance is improved. PCH cells also show improved SET speed and lower programming energy than GeSbTe‐based cells, which stems from the faster crystallization of Sb_2_Te_3_, the thinness of the active layers and the ability of TiTe_2_ to act as thermal barrier due to its low thermal conductivity, effectively suppressing the vertical heat loss during programming.

While these results are promising, there is still considerable room for improvement by employing carefully designed CM/PCM material combinations. For instance, PCHs based on pure Sb_2_Te_3_ suffer from inadequate data retention for some applications, due to insufficient stability of the amorphous phase. In this computational work we consider superlattice PCHs containing TiTe_2_ as CM and Ge_2_Sb_2_Te_5_ (GST‐225) or GeTe as PCMs, which are known to have a very stable glassy state at ambient conditions.

## 2 Crystalline PCHs

**Figure 1:**
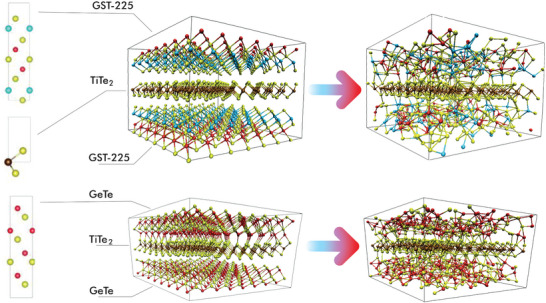
The structures to the left of the arrow are the crystalline models of GST‐225/TiTe_2_ (upper) and GeTe/TiTe_2_ (lower) PCHs after geometry optimization at *T* = 0 K. On the very left the crystalline building blocks used to construct the PCH models are depicted. Starting from top to bottom the GST‐225, TiTe_2_ and GeTe blocks are shown, respectively. The structures to the right of the arrow are the partially melted models of GST‐225 and GeTe/TiTe_2_ at *T* = 1300 K. We depict Te, Ti, Ge, and Sb atoms in yellow, brown, red, and cyan, respectively.

Our analysis begins by examining the structural properties of the fully crystalline PCHs at 0 K. Since we employ density‐functional‐theory (DFT) codes based on periodic boundary conditions, we investigate superlattice heterostructures that exhibit periodicity along the vertical direction as well. To minimize the lattice in‐plane mismatch between the PCM and the CM building blocks, we utilize large supercells (SC). **Table** **1** showcases the size of the SCs and the percentage of the in‐plane strain of the PCMs. Our models have a single trilayer of TiTe_2_ acting as the CM. The GST‐225 slab comprises 441 atoms arranged in nine atomic layers, corresponding to the basic building block of the hexagonal phase. It is stacked as in the Kooi‐De Hosson phase,^[^ ^16^ ^]^ namely Te–Sb–Te–Ge–Te–Ge–Te–Sb–Te. The GeTe slabs contain 512 atoms arranged in four bilayers. The direction perpendicular to the bilayers corresponds to the [111] direction of the trigonal, ferroelectric *α* phase of GeTe.^[^ ^17^ ^]^ Spatial gaps separate the CM and PCM blocks, as shown in **Figure** **1**. We refer to these gaps as van der Waals (vdW) gaps.

**Table 1:** SC lattice parameters of the two PCHs after cell optimization at *T* = 0 K. *a*, *b*, and *c* represent the module of the SC lattice vectors. The parameters *α*, *β* and *γ* are the SC angles. ”Replicas” indicates the number of PCM (CM) building blocks considered to minimize the lattice mismatch between the CM and PCM blocks. We also indicate the total number of atoms in each PCH and the percentage of in‐plane strains. All the distances are expressed in Å  units.

	GST‐225/TiTe_2_	GeTe/TiTe_2_
*a*	30.07	33.56
*b*	30.07	33.56
*c*	24.86	22.41
*α*	90°	90°
*β*	90°	90°
*γ*	120°	120°
Replicas	7 × 7 (8 × 8)	8 × 8 (9 × 9)
Tot atoms	633	755
Strain	1%	0.3%

We use the QUICKSTEP code in the CP2K package^[^ ^18^ ^]^ to optimize the internal structure and the lattice parameters of the PCHs. To that aim, we also use scalar‐relativistic Goedecker‐Teter‐Hutter (GTH) pseudopotentials.^[^ ^19^ ^]^ In Section S1 (Supporting Information) we report all the computational details. In Figure 1, we present the two relaxed heterostructures and in Table 1 their resulting lattice parameters.

It is well known that pure Perdew‐Burke‐Ernzerhof (PBE) calculations yield too large vdW gaps in bulk GST‐225 and TiTe_2_ and, thus, too large lattice parameters *c*. To accurately depict the chemical interactions in the vdW gaps, here we complement the PBE functional with Grimme‐D3 (PBE‐D3) vdW corrections.^[^ ^20^ ^]^ First, we calculate the theoretical equilibrium lattice parameters of the two bulk models at 0 K by performing cell relaxation. For GST‐225, we obtain: *a* = 4.32 Å  and *c* = 17.50 Å  (PBE) and *a* = 4.35 Å  and *c* = 17.05 Å  (PBE‐D3), to be compared with the experimental data at 300 K^[^ ^16^ ^]^: *a* = 4.2 Å  and *c* = 17.2 Å . Taking into account that the coefficient of thermal expansion of GST‐225 at 200 °C is equal to 1.74 × 10^−5^K^−1^ (see ref. [21]), the lattice parameters at ambient temperature should differ from the 0 K values by at most 1 %. Thus, the PBE‐D3 *c* parameter is in very good agreement with the experimental one (there are minor discrepancies concerning the *a* parameter but this is somewhat less important, since a small planar strain was induced in our superlattice models to match it with that of the CM). With regards to TiTe_2_, PBE‐D3 provides an excellent description of its structural properties, in contrast to pure PBE. In fact, our simulations yield: *a* = 3.81 Å  and *c* = 6.74 Å  (PBE) and *a* = 3.79 Å  and *c* = 6.56 Å  (PBE‐D3), while the experimental parameters are ref. [22]: *a* = 3.78 Å  and *c* = 6.5 Å  (PBE). We note that the PBE‐D2 correction^[^ ^23^ ^]^ gives similar lattice parameters as PBE‐D3 for these two layered compounds (see Supporting Information). Since, we study not only crystalline PCHs but also partly liquid or amorphous structures with disordered interfaces, we choose the D3 correction because it uses structure‐dependent dispersion coefficients, which should ensure wider applicability.

After the lattice parameters' optimization, the two PCHs display significant differences near the vdW gaps. The GST‐225/TiTe_2_ PCH exhibits nearly perfect gaps, with a weak chemical interaction of 108 meV per atom between the Te atoms on the two sides of the vdW gap. This behavior is due to the layered structure of both GST‐225 and TiTe_2_. In contrast, the interaction at the interfaces between the GeTe and TiTe_2_ blocks is stronger (182 meV per atom), and the atomic planes near the gaps show non‐negligible corrugation (first two columns of **Table** **2**). This fact is also expected, as GeTe is a “three‐dimensionally bonded” material,^[^ ^24^, ^25^ ^]^ hence the interface layers of the GeTe block should be more reactive than those of the GST‐225 block. Furthermore, in the GeTe/TiTe_2_ model, the two vdW gaps are structurally and chemically asymmetric: one consists of two Te planes, whereas the second one includes a Te plane facing a Ge plane.

**Table 2:** Thicknesses $L_{GAP}^{\left(1\right)}$ and $L_{GAP}^{\left(2\right)}$ of the first and second vdW gap at *T* = 0 K (crystalline PCM), 600 K (crystalline PCM), 1300 K (liquid PCM) and 300 K (amorphous PCM) in the two PCHs. As regards the GeTe‐based structure, $L_{GAP}^{\left(1\right)}$ refers to the Te‐Te interface, while $L_{GAP}^{\left(2\right)}$ refers to the Ge‐Te interface. All the thicknesses are expressed in Å units.

	0 K (crystalline)		600 K (crystalline)		1300 K (liquid)		300 K (amorphous)	
	GST‐225	GeTe	GST‐225	GeTe	GST‐225	GeTe	GST‐225	GeTe
$L_{GAP}^{\left(1\right)}$	3.43	3.37	3.38	3.23	3.18	3.13	3.48	3.25
$L_{GAP}^{\left(2\right)}$	3.45	2.72	3.36	2.73	3.16	3.18	3.23	3.36

Subsequently, we conduct ab initio molecular dynamics (AIMD) simulations in the canonical ensemble (NVT) employing Born‐Oppenheimer molecular dynamics and the second‐generation Car‐Parrinello molecular dynamics developed by Kühne et al.^[^ ^26^ ^]^ We describe the AIMD simulations' protocol in Section S2 (Supporting Information). We set the temperature to 600 K, which is lower than the melting points of bulk GST‐225, bulk GeTe (900 and 998 K, respectively) and bulk TiTe_2_ (1470 K). Despite the system remains crystalline, the vdW gaps decrease due to thermal disorder, as noted in Table 2. The vdW gaps in the GST‐225 structure shrink and remain spatially symmetric on average. However, in the GeTe‐based structure the increase in temperature dose not reduce the gap of the Ge‐Te interface due to the presence of relatively strong chemical interaction. In Section S3 (Supporting Information), we provide a detailed analysis of the temperature‐induced changes in the vdW gaps.

## 3 Partial Melting

We carry out AIMD simulations of melting of the confined PCMs in order to study the RESET process characterizing the devices based on PCHs. In this way, we also assess the stability of the superlattice structure at temperatures higher than the melting temperatures of the two PCMs. We find it particularly interesting to observe the behavior of the GeTe/TiTe_2_ PCH as it displays significant chemical interaction across the vdW gaps in the crystalline state.

As we describe in Section S2 (Supporting Information), we raise the temperature from 600 to 1700 K and we run a 10 ps simulation at this temperature to rapidly achieve melting. We underline that the crystalline structure of the CM remains stable during this short run, even though the temperature is higher than its melting temperature. Afterward, we decrease the temperature to 1300 K, which leads to the complete melting of the PCMs in about 70 ps. In Figure 1, we show a snapshot of these phases, where the liquid‐disordered structure of the confined PCMs is evident along with the crystalline structure of the CM.

At *T* = 1300 K, we observe further changes in the vdW gaps. In GST‐225/TiTe_2_ PCH the thickness of the vdW gaps decreases while in the GeTe/TiTe_2_ PCH, the two vdW gaps become more symmetrical than those observed at *T* = 0 and 600 K (see Table 2). Furthermore, in the two regions of the GeTe slab near the vdW gaps, we find a concentration of Te atoms above 50%. Specifically, we define these two regions based on the widths of the outermost peaks of the atomic density profiles *ρ*(*z*) (discussed in more details in Section IIIB and Section S3 of Supporting Information). The average populations obtained are 103 Te atoms (40.6% of the total Te atoms in the GeTe slab) and 74 Ge atoms (32.8%), corresponding to a local stoichiometry of Ge_4.18_Te_5.82_. The latter finding is also reported in ref. [27], where a superlattice consisting of switching GeTe layers and artificial slabs of CMs made of frozen bilayers of crystalline GeTe is considered. As regards the GST‐225/TiTe_2_ structure, the PCM regions facing the gaps have only a slight excess of Te atoms, corresponding to the stoichiometry Ge_1.78_Sb_1.84_Te_5_: the average populations are 87 Te atoms (35.1%), 31 Ge atoms (22.4%), and 32 Sb atoms (32.7%).

### 3.1 Structural Properties

After thermal equilibration, we analyze the structural properties of the two PCHs at *T* = 1300 K by computing various correlation functions. First, we consider the total radial distribution function (RDF) *g*(*r*), defined as:

(1) $$g\left(r\right)=\frac{V}{N^{2}}\langle \sum_{i}\sum_{j\neq i}\delta \left(r-r_{ij}\right)\rangle$$

In Equation (1), *N* represents the number of atoms in the system, *V* is the SC volume, *i* and *j* are atomic indexes, and the brackets denote an average over all particles and configurations generated with the AIMD simulations.

**Figure 2:**
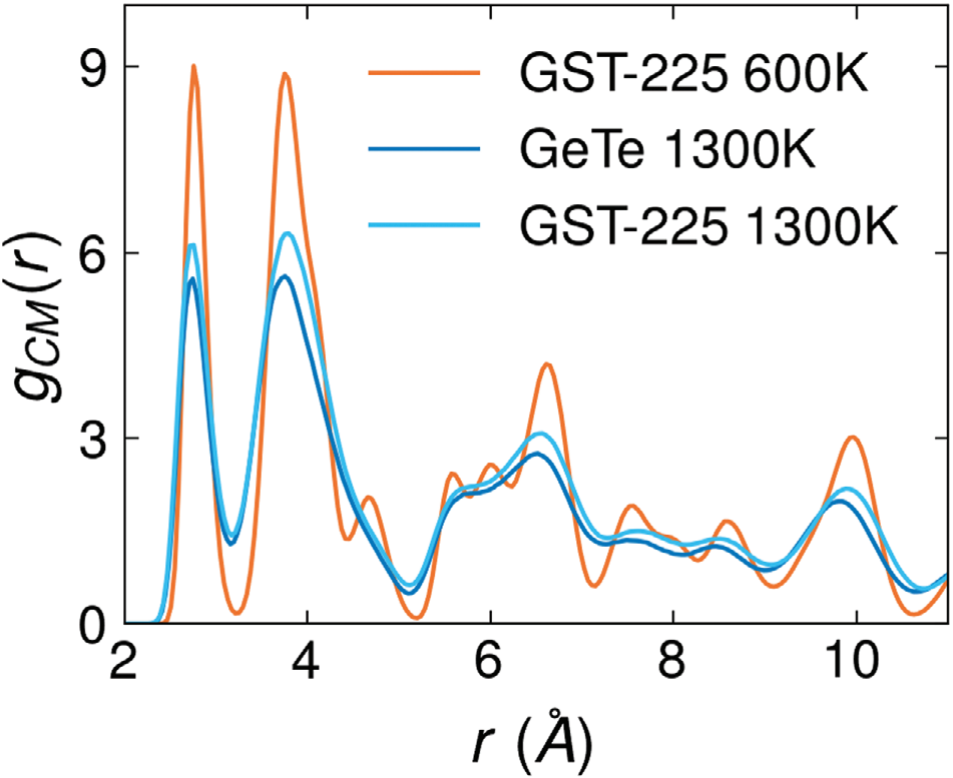
*g*(*r*) of the CM (TiTe_2_) inside the two PCHs at *T* = 600 K and *T* = 1300 K. The orange curve referring to the CM in the GST‐225/TiTe_2_ structure at *T* = 600 K is basically indistinguishable to the one relative to the GeTe/TiTe_2_ PCH at *T* = 600 K (not shown). The blu and cyan curves refer to the GeTe/TiTe_2_ and GST‐225/TiTe_2_ structures at *T* = 1300 K.

In **Figure** **2**, we report the TiTe_2_ RDFs for the two PCHs at *T* = 600 K and *T* = 1300 K. It clearly shows that the TiTe_2_ trilayer remains in its crystalline state throughout the process described in the previous Section. At *T* = 1300 K, the RDF's peaks appear slightly broader due to increased thermal motion of the CM atoms. Hence, we can conclude that the partial‐melting process does not have a destabilizing effect on the two PCHs within the time scales that are accessible by our simulations.

Our investigation continues with the analysis of the confined liquid PCMs (*l*‐PCMs) in the heterostructures. We carry out a comparative analysis of their total *g*(*r*) and partial radial distribution functions (pRDFs) *g_*αβ*_(r*), where *α* and *β* represent atomic species, with those of their corresponding bulk models that we derive from separate AIMD simulations. We construct the crystalline SCs of the bulk models by periodically repeating along the *c* direction the crystalline PCM slabs taken from the corresponding PCHs. Then we melt down these systems following the same protocol used for PCH models and reported in Section S2 (Supporting Information).

We analyze the various pRDFs of the confined PCMs to check that they are indeed in the liquid phase. Then, we analyze their structural features against their bulk equivalents. By pointing out that *dn*_*αβ*_(*r*) is the number of atom pairs (*α*, *β*) at a distance ranging from *r* to *r* + *dr* and *ρ_β_* is the atomic density of *β* species we can define a pRDF *g*_*αβ*_(*r*) as:

(2) $$g_{\alpha \beta }\left(r\right)=\frac{1}{\rho_{\beta }}\frac{dn_{\alpha \beta }\left(r\right)}{4\pi r^{2}dr}$$

During our investigation, we utilize numerical differentiation to obtain the first maxima $\bar{r}$ and minima *r*_*cut*_ of the various pRDFs. These values correspond to the most probable atomic bond lengths and the size of the first coordination shell, respectively. If a pRDF does not exhibit any minimum up to 3.5 Å, we set *r*_*cut*_ to this value. We note that the RDFs and pRDFs of the confined and bulk *l*‐PCMs cannot be directly compared because of the different SC volumes involved. However, it is meaningful to compare the partial and total coordination numbers (CNs). To find the partial CNs *N*_*αβ*_, we integrate the pRDFs up to *r*_*cut*_, as per Equation (3):

(3) $$N_{\alpha \beta }=4\pi \rho_{\beta }\int_{0}^{r_{cut}}\;\;\;\;\;g_{\alpha \beta }\left(r\right)\;r^{2}dr$$

**Figure 3:**
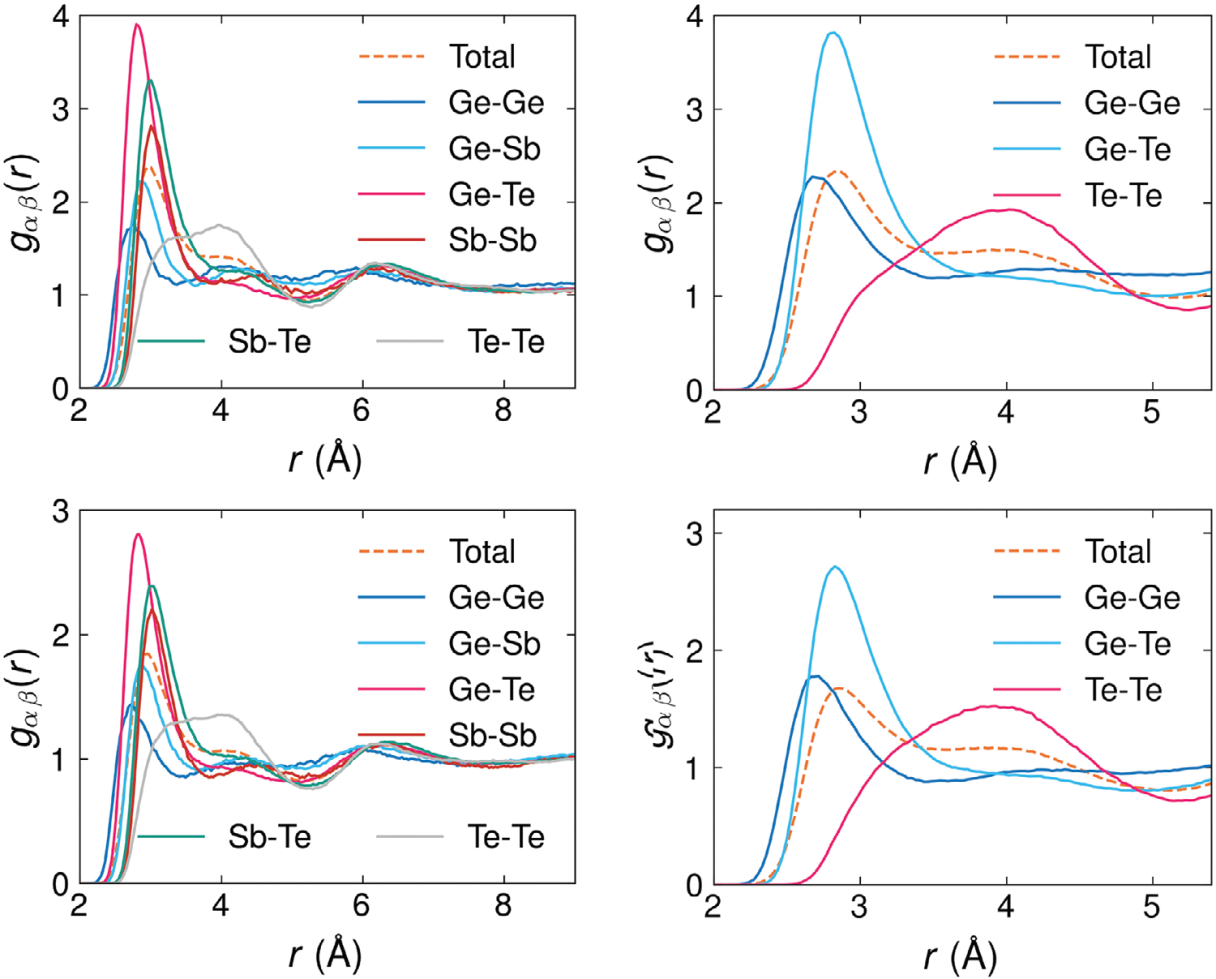
The left plots depict the pRDFs (solid lines) and total (dashed line) RDF of *l* −GST‐225 at *T* = 1300 K in the confined (upper plot) and bulk (lower plot) cases. The right plots depict the pRDFs (solid lines) and total (dashed line) RDF of *l* −GeTe at *T* = 1300 K in the confined (upper plot) and bulk (lower plot) cases.

All the *r*_*cut*_ values are listed in **Table** **3**. From Equation (3) we can define the total CN for atomic species *α* as *N*_*α*_ = ∑_*β*_ *N*_*αβ*_. We provide the pRDFs of confined and bulk *l*‐GST‐225 and *l*‐GeTe in **Figure** **3**. Additionally, we list all their total and partial CNs in **Table** **4**.

**Table 3:** First maxima ($\bar{r}$) and first minima (*r*_*cut*_) of the pRDFs of the confined *l*‐GST‐225 and *l*‐GeTe and their bulk counterparts at *T* = 1300 K. All the distances are expressed in Å  units.

	GST‐225		GeTe	
	Conf	Bulk	Conf	Bulk
${\bar{r}}_{Ge-Ge}$	2.75	2.77	2.7	2.7
*r*_*cut*_^*Ge* − *Ge*^	3.4	3.4	3.4	3.4
${\bar{r}}_{Ge-Te}$	2.8	2.86	2.8	2.8
*r*_*cut*_^*Ge* − *Te*^	3.5	3.5	3.5	3.5
${\bar{r}}_{Te-Te}$	4.0	4.0	4.0	4.0
*r*_*cut*_^*Te* − *Te*^	3.5	3.5	3.5	3.5
${\bar{r}}_{Ge-Sb}$	2.9	2.89	/	/
*r*_*cut*_^*Ge* − *Sb*^	3.5	3.5	/	/
${\bar{r}}_{Sb-Sb}$	3.02	3.04	/	/
*r*_*cut*_^*Sb* − *Sb*^	3.5	3.5	/	/
${\bar{r}}_{Sb-Te}$	3.01	3.07	/	/
*r*_*cut*_^*Sb* − *Te*^	3.5	3.5	/	/

**Table 4:** Average partial and total CNs of the *l*‐PCM atoms at *T* = 1300 K. The word “Bulk” refers to the bulk models, whereas “Conf” (“Conf+”) refers to the *l*‐PCM slabs in the PCH excluding (including) the Te atoms of TiTe_2_.

GST‐225 (1300 K)									
	*N*_*Ge* − *Ge*_	*N*_*Ge* − *Sb*_	*N*_*Ge* − *Te*_	*N*_*Sb* − *Sb*_	*N*_*Sb* − *Te*_	*N*_*Te* − *Te*_	*N*_*Ge*_	*N*_*Sb*_	*N*_*Te*_
Bulk	0.81	0.98	3.61	1.06	3.10	1.80	5.40	5.14	4.48
Conf	0.74	0.88	3.53	1.00	3.04	1.67	5.15	4.92	4.30
Conf+	0.74	0.88	3.78	1.00	3.21	1.68	5.40	5.09	4.48

GeTe (1300 K)									
Bulk	2.36	/	3.48	/	/	1.54	5.84	/	5.02
Conf	2.13	/	3.24	/	/	1.29	5.37	/	4.53
Conf+	2.13	/	3.51	/	/	1.39	5.64	/	4.90

We find that, in all cases, the main features of the RDFs and pRDFs for the confined and bulk *l*‐PCMs are similar, including the positions of the maxima. Moreover, the bulk *l*‐GST‐225 and bulk *l*‐GeTe *g*(*r*)'s show almost identical behavior to the one reported in ref. [28, 29].

As far as confined *l*‐GST‐225 is concerned, the absolute maxima of the *g*_*αβ*_(*r*)'s fall within the range of 2.75 to 3 Å  for most pairs. In particular, Ge‐Ge, Ge‐Te, and Ge‐Sb pRDFs show peaks at 2.75 , 2.8,  and 2.9 Å, and their magnitude indicates the presence of a sizable number of such bonds. On the contrary, the Te‐Te pRDF has a broad peak around 4 Å, which is slightly more prominent than the one of the bulk system. The Ge‐Te and Sb‐Te pRDFs display a shoulder at distances exceeding 3.5 Å  rather than a minimum. The first minimum is observed for all other pairs at distances greater than 3.5 Å, except for the Ge‐Ge case, where the minimum is located at 3.4 Å. Consequently, the total RDF shows a first peak centered at 3 Åwith a shoulder around 4 Å. This trend aligns with that reported in ref. [28].

In confined *l*‐GeTe, the Ge‐Ge and Ge‐Te pRDF exhibit their first maxima at 2.7  and 2.8 Å. The second value is comparable to the short Ge‐Te bond length of *α*‐GeTe at room temperature, where each Ge and Te atom has three shorter (2.8 Å) and three longer (3.2 Å) bonds.^[^ ^17^, ^30^, ^31^ ^]^ The Te‐Te pRDF main peak is centered around 4 Å  and envelops the entire second coordination shell in this model too. As a result, the total RDF of *l*‐GeTe displays a first peak at smaller radii but a shoulder at similar radii in comparison with the *l*‐GST‐225 RDF. This trend is consistent with the findings of ref. [29].

Table 4 lists all the CNs obtained by means of Equation (3). There the wording “Conf+” refers to the case where the Te atoms of the CM are included for the calculation of the CNs. In the latter case, the values of *N*_*X* − *Te*_, where *X* stands for Ge, Sb, or Te, increase slightly but not significantly, indicating weak interaction between the confined *l*‐PCMs and the CM even at 1300 K. We notice slightly lower CNs for the confined *l*‐PCMs compared to the bulk case. Table 4 shows that in confined *l*‐GST‐225, Ge and Sb mainly form bonds with Te. The total and partial CNs for bulk *l*‐GeTe and *l*‐GST‐225 are larger than those reported in refs. [28, 29] for *T* = 1250 K. The primary reason for this discrepancy is the choice of the cutoff radii, which, in those works, are smaller than the ones in Table 3. In Section S4 of (Supporting Information) we provide the CNs as a function of the radius *r*_*cut*_: using the same values of refs. [28, 29], we obtain similar CNs to the ones indicated in those works, except for the Te‐Te case. The remaining differences can be ascribed to the slightly different volumes involved (for instance, when constructing the bulk GST‐225 SC, we choose the theoretical equilibrium density of 0.031 atomÅ^−^3 at *T* = 0 K, whereas in ref. [28, 29] the experimental value of 0.030 atomÅ^−3^ is selected) and the use of a different method to describe vdW interactions (D3 correction vs. ab initio DF2^[^ ^32^ ^]^).

**Figure 4:**
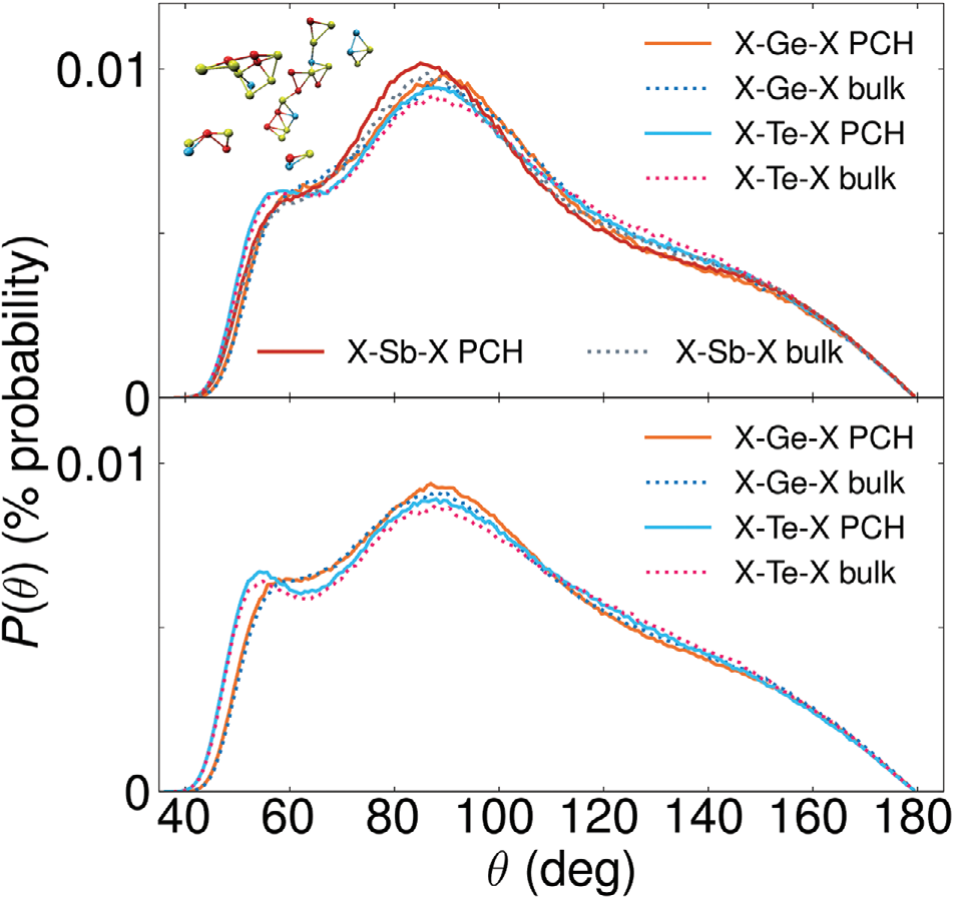
Sub‐ADFs of conf (solid lines) and bulk (dotted lines) *l*‐GST‐225 (upper plot) and *l*‐GeTe (lower plot) at *T* = 1300 K. The inset of the upper plot shows typical triangular structures in *l*‐GST‐225 responsible for the appearance of a shoulder at 55°. A similar picture is valid for the *l*‐GeTe case but is not shown.

To further investigate the structural properties of confined *l*‐PCMs, we calculate their angular distribution functions (ADFs) *P*(θ). These functions describe the distribution of the angles enclosed by specific triplets of atoms. We use the term “sub‐ADF” when referring to the ADFs resolved for different central atoms. We analyze the sub‐ADFs of the two confined *l*‐PCMs and compare them with their bulk counterparts in **Figure** **4**. We observe that there are no significant differences between the confined and bulk *l*‐PCMs for any environment: a broad peak always appears prominently at approximately 90°, and an additional shoulder is always present at about 55°. The range of angles covered by the peak and the average CNs in Table 4 suggest predominant defective octahedral environments.

In Section S4.2 (Supporting Information), we show the partial ADFs (pADFs) of the confined *l*‐PCMs resolved over different triplets of atomic species. It turns out that in both *l*‐GST‐225 and *l*‐GeTe all types of triplets contribute to the principal peak around 90° and the broad tail exceeding 110° angles. In both PCMs we observe a shoulder at 55° that corresponds to edge‐sharing “triangles”, some of which are shown in the upper left part of Figure 4. None of the Te − • − Te bond configurations contributes significantly to the shoulder in the confined models. We also study the tail of the ADFs spanning from 155° up to 180°. In particular, we investigate almost‐aligned‐atomic triplets with inter‐bond angles in the range between 155°‐180° with the help of angular‐limited three‐body correlation functions (ALTBCs) *g*_3_(*r*_*AB*_, *r*_*BC*_):

(4) $$g_{3}\left(r_{AB},r_{BC}\right)=\frac{1}{N}\sum_{i,j,k}\langle \delta \left(r_{AB}-r_{ij}\right)\delta \left(r_{BC}-r_{ik}\right)\Theta \left(\beta +\gamma \right)\rangle$$

where Θ is the Heaviside function, *γ* ∈ (0, 25°), *β* = π − *α, α* is the bond angle, $N={\left(4\pi \right)}^{2}r_{AB}^{2}r_{BC}^{2}$ is the normalization factor and the brackets denote an average over all the configurations of the AIMD simulations. The ALTBC indicates how likely it is to find an atomic triplet whose central atom, denoted as *B*, has one bond of length *r*_*AB*_ almost aligned with a second bond of length *r*_*BC*_. Therefore, the ALTBC allows to detect the presence of Peierls‐like distortions, which consist of alternating short and long bonds on the opposite sides of an atom. Such distortions are reminiscent of the genuine Peierls distortions that occur in the *α* phase of GeTe.

**Figure 5:**
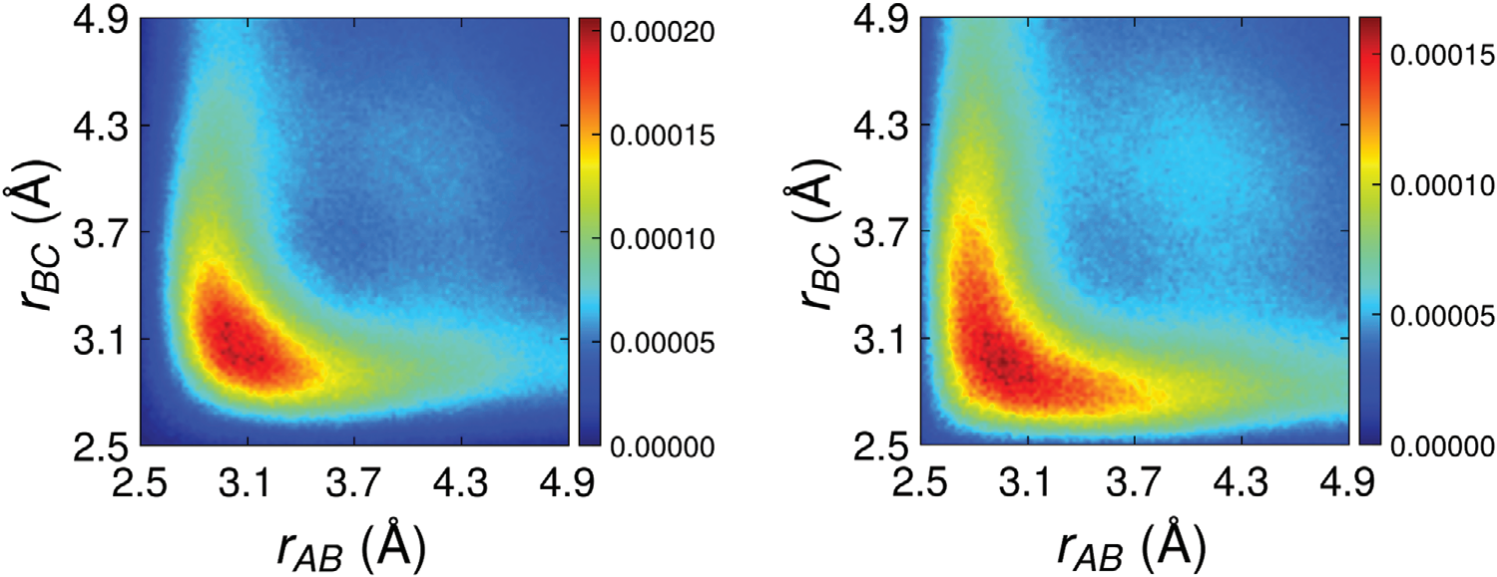
Total ALTBC of confined *l*‐GST‐225 (left plot) and *l*‐GeTe (right plot) at *T* = 1300 K.

In **Figure** **5**, we show the total ALTBCs of the two *l*‐PCMs. All the plots exhibit a single broad peak centered on the diagonal (*r*_*AB*_ = *r*_*BC*_), indicating that the majority of atomic triplets in confined *l*‐PCMs do not show Peierls‐like distortions. The same is true for Ge‐, Te‐ and Sb‐centered sub‐ALTBCs for both systems. However, the confined *l*‐GeTe (lower plot) shows a more stretched peak. The ALTBC plots of the two bulk *l*‐PCMs do not show distortions either (Section S4.3, Supporting Information). Our results appear to be in contrast with refs. [28, 29], where two peaks off the diagonal corresponding to Peierls‐like distortions appear for bulk *l*‐GeTe and *l*‐GST‐225 at temperatures up to 1250 K. We attribute the discrepancy to the use of the DF2 vdW functional in refs. [28, 29].

To complete the analysis of local structures, we examine the distributions of the Errington‐De Benedetti local order parameter^[^ ^33^ ^]^ *q*, which provides information on the local environment of an atom. In particular, it quantifies the presence of tetrahedral and octahedral configurations (defective or not). The order parameter *q* for an atom *k* is defined as:

(5) $$q\equiv q_{k}=1-\frac{3}{8}\sum_{i=1}^{N_{k}^{\left(b\right)}-1}\sum_{j\neq i+1}^{N_{k}^{\left(b\right)}}{\left(\frac{1}{3}+\cos\theta_{ijk}\right)}^{2}$$

where θ_*ijk*_ is the angle formed by the lines connecting the atom *k* and its nearest neighbors *i* and *j* and the sum runs over all pairs (*i*, *j*) of atoms $i,j=1,\cdots ,N_{k}^{\left(b\right)}$ bound to a central atom *k*. As shown in Section S4.4 (Supporting Information), we found that in *l*‐GST‐225 Ge and Sb are primarily four,five and sixfold coordinated, while Te is three,four and fivefold coordinated. Similarly, in *l*‐GeTe Ge has coordination 4,5, and 6 and Te is mainly three, four, and five‐fold coordinated. However, we observe an increase in the number of sixfold coordinated Te atoms with respect to *l*‐GST‐225. In presence of fourfold coordination *q* = 1 identifies perfect tetrahedral coordination, while *q* = 0 and sixfold coordination corresponds to a perfect octahedral structure. Defective octahedral geometries are represented by values in the range of (− 3, 1). It turns out that in the two confined *l*‐PCMs, tetrahedral Ge atoms are almost absent (2.6% in *l*‐GST‐225 and 1.1% in *l*‐GeTe) while various types of defective octahedral structures are present, including: threefold pyramidal, fourfold planar and non‐planar defective octahedral, and fivefold defective octahedral structures.

### 3.2 Diffusion Coefficients

To determine the self‐diffusion coefficient *D*_*z*_ in the vertical direction (referred to as the *z*‐direction) for the two PCMs at *T* = 1300 K, we utilize Einstein's formula:

(6) $$D_{z}=\frac{1}{2}\lim_{t\to \infty }\frac{d}{dt}\langle {\text{MSD}}_{z}\left(t\right)\rangle$$

where MSD_*z*_ denotes the mean square displacements of the *l*‐PCMs' atoms along the *z* direction. Here, the notation 〈 · 〉 indicates an average over the trajectory of each atom throughout the AIMD simulations. To obtain a reliable value for *D*_*z*_, we fit the averaged MSD_*z*_(*t*) using a linear function in the range where it exhibits a linear profile with respect to time. More specifically, the function MSD_*z*_(*t*) is defined as:

(7) $${\text{MSD}}_{z}\left(t\right)=\frac{1}{T_{run}-t}\sum_{i=0}^{T_{run}-t}{\left[z\left(i+t\right)-z\left(i\right)\right]}^{2}$$

where *T*_*run*_ designates the length of the entire AIMD simulation, *z* is the vertical position of a particle and *i* and *t* are time lag indexes. In Equation (6), the ensemble average is evaluated by averaging all contributions to the MSD in Equation (7) that refer to the same time lag.

The formula in Equation (7) can be straightforwardly used to analyze the global *D*_*z*_ value of the *l*‐PCMs, both in the confined and the bulk cases. **Table** **5** shows the resulting values of *D*_*z*_. Confinement leads to smaller average diffusion coefficients. Moreover, we observe differences in the *D*_*z*_ values of the individual atomic species in *l*‐GeTe and *l*‐GST‐225 (see Table 5). In agreement with previous work,^[^ ^34^ ^]^ we find that Te has lower diffusion coefficient than Ge in both models of GeTe. However, Sb turns out to be the least mobile species in our bulk model of GST‐225, contrary to ref. [34]. On the other hand, Sb is the most mobile species in the confined model of GST‐225. We observe that confinement leads to a reduction in the mobility of Te atoms to less than half of the bulk value. The reason for this decrease is a layering effect in the liquid PCM induced by the interface, as discussed in Section S5 (Supporting Information).

**Table 5:** Comparison of *D*_*z*_ values for the two l‐PCMs in bulk and confined cases at *T* = 1300 K. We resolved *D*_*z*_ over the single atomic species.

	$D_{z}\;\times 10^{-10}m^{2}s^{-1}$			
	GST‐225 bulk	GST‐225 conf	GeTe bulk	GeTe conf
Ge	44.51	26.27	48.41	25.26
Te	39.24	18.84	32.63	17.73
Sb	37.61	28.53	/	/
global	40.05	22.65	40.52	21.49

**Figure 6:**
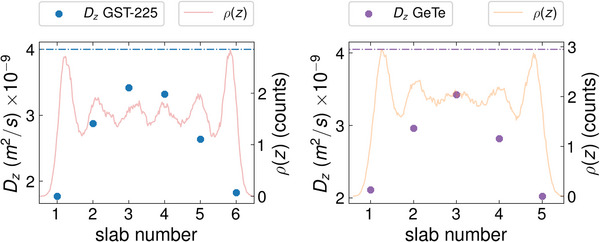
Profile of the *D*_*z*_ self‐diffusion coefficient in the PCM region for the two confined *l*‐PCMs at *T* = 1300 K. These regions are divided into slabs and the *D*_*z*_ values are given for each slab. The *l*‐GeTe PCH has fewer slabs (five) than the PCH containing *l*‐GST‐225 (six). The dash‐dotted lines refer to the $D_{z}^{\left(bulk\right)}$ values listed in Table 5 and employed in Equation (8). The left and right plot refer to *l*‐GST‐225 and *l*‐GeTe, respectively. We also report the atomic density profiles *ρ*(*z*) of the two confined *l*‐PCMs at *T* = 1300 K along the *z* direction. The *l*‐GST‐225 and *l*‐GeTe *ρ*(*z*) are shown as red and orange shaded curves, respectively. The *z* values increase as the slab number increases (the *z* axis is not shown). The peaks of the *ρ*(*z*) profiles define the size of the slabs inside which the corresponding *D*_*z*_ coefficients are calculated.

To gain a more thorough understanding of the impact of confinement and interface effects on the diffusion, we also compute the *D*_*z*_ value of the confined *l*‐PCMs as a function of the distance from the vdW gaps. For this purpose, some modifications to the algorithm need to be made. First, we exclude the region occupied by the CM, which retains its crystalline features, as shown in Figure (2), with vanishing self‐diffusion coefficient. Then, we partition the region occupied by the *l*‐PCM into multiple slabs parallel to the *XY* plane. The slicing is made based on the atomic density profiles *ρ*(*z*) shown in **Figure** **6**: each slab is defined as the volume enclosed between two density minima. We obtain six slabs in the case of *l*‐GST‐225 and five slabs for *l*‐GeTe. The resulting slabs have varying thickness, as evidenced in Figure 6: for *l*‐GST‐225 the thickness is 4Å  for the slabs close to the CM and 3Å  for the others; for *l*‐GeTe the very central slab and the two external ones are 4Å  wide, the remaining slabs are 3 Å  wide. We define the surviving time *t*_*surv*_ in a slab as the average time needed by an atom to diffuse across the slab:

(8) $$t_{surv}=\frac{{\left(\Delta z\right)}^{2}}{2D_{z}^{\left(bulk\right)}}$$

In Equation (8), $D_{z}^{\left(bulk\right)}$ is the value of the self‐diffusion coefficient along the *z* direction of the bulk *l*‐PCM computed from AIMD simulations (shown in Table 5) and Δ*z* is the height of the slab. Initially, we considered different *t*_*surv*_ values since the slabs have different heights. In the end, we use the values obtained by setting Δ*z* = 3Å  even for thicker slabs, since the discrepancies are negligible. Equation (8) defines the time window in which the MSD_*z*_(*t*) of each slab is to be repeatedly analyzed. For each slab, a MSD_*z*_(*t*) averaged over all atoms is calculated for successive time windows lasting *t*_*surv*_ until covering the total simulation time. Subsequently, we take an average over all time windows and we obtain the *D*_*z*_ of a single slab. More information is provided in Section S5 (Supporting Information).

Confinement makes the *D*_*z*_ of the *l*‐PCMs to be larger in their central region, farther away from the CM, while at the interface with the CM *D*_*z*_decrease toward zero, as shown in Figure 6 and also reported in Table S3 of Section S5 (Supporting Information). The *D*_*z*_ profiles are lower than the corresponding bulk values listed in Table 5 (which are represented as dash‐dotted lines in Figure 6) even in the center of the slabs due to finite size effects. Throughout the entire MD simulations at 1300 K running for more than ≈50 ps, no PCM atom penetrates the CM trilayer. The PCM atoms diffuse close to the boundaries, but all bounce back: the CM acts as a barrier against the migration of *l*‐PCM atoms along the *z* direction. This phenomenon also determines the shape of *ρ*(*z*) for each PCH: the atoms of the *l*‐PCMs cluster at the edges of the PCM blocks. Our results are in line with the simulations of Sb_2_Te_3_/TiTe_2_ PCHs presented by Ding et al. in ref. [15].

## 4 Partial Amorphization

We amorphize the PCM portion of the two PCHs by quenching the models down to 300 K with the following protocol: from 1300 to 900 K the quenching rate is 10^15^ Ks^‐1^, whereas it is set to 10^13^ Ks^‐1^ from 900 to 300 K. Before the second quenching, the system is briefly equilibrated for 5 ps at 900 K. We show snapshots of both amorphous models in Section S6 (Supporting Information).

The GST‐225/TiTe_2_ model displays slightly asymmetric vdW gaps (see Table 2): one of them has a thickness comparable to those of the gaps of the fully crystalline models, whereas the second one is about 7% thinner. The two regions of the GST‐225 slab near the interfaces (which are defined by the corresponding peaks of the *ρ*(*z*) profiles shown in Section S3, Supporting Information) have the following average populations: 11 Ge atoms, 18 Sb atoms, 44 Te atoms ($L_{GAP}^{\left(1\right)}$) and 11 Ge atoms, 14 Sb atoms, 42 Te atoms ($L_{GAP}^{\left(2\right)}$). Thus, the wider gap is characterized by a lower relative concentration of Ge atoms. Summing up the contribution of the two interfaces, we obtain: 22 Ge atoms (22.4% of total Ge atoms), 32 Sb atoms (32.7%), and 86 Te atoms (35.1%), which correspond to a local stoichiometry of Ge_1.28_Sb_1.86_Te_5_. Concerning GeTe/TiTe_2_, the two gaps are instead more symmetric than in the crystalline models, since in the latter the two interfaces are chemically very different. Analogously to the partly liquid GeTe/TiTe_2_ model, there is an excess of Te atoms in the interface regions of the amorphous GeTe slab. These regions are populated by an average of 104 Te atoms (40.6%) and 84 Ge atoms (32.8%), corresponding to Ge_4.47_Te_5.53_.

### 4.1 Structural Properties

**Figure 7:**
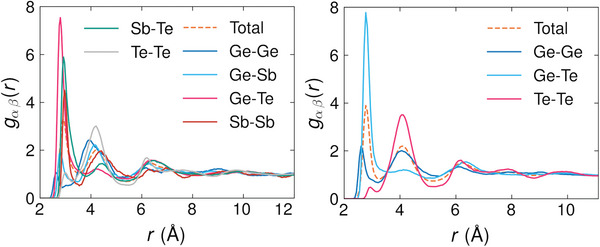
pRDFs (solid lines) and total RDFs (dashed lines) of a‐GST‐225 (left plot) and a‐GeTe (right plot) at *T* = 300 K.

**Figure** **7** displays the RDFs and pRDFs of amorphous GST‐225 (a‐GST‐225) and amorphous GeTe (a‐GeTe) at a temperature of 300 K. When referring to confined amorphous PCM, we will simply write a‐PCM, since no bulk counterparts are considered in this part of the work.

**Figure 8:**
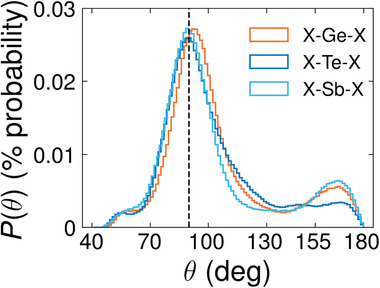
sub‐ADFs of a‐GST‐225 at *T* = 300 K. The black dashed vertical line is located at θ = 90°.

The a‐GST‐225 RDF exhibits three distinct peaks, with the first peak located around 2.92 Å  representing the average distance between nearest neighbors. Our analysis indicates that first‐neighbor pairs primarily consist of Ge‐Te (peak at 2.86 Å) and Sb‐Te (peak at 2.92 Å). However, we also observed Sb‐Sb pairs (peak at 2.96 Å) and, to a lesser extent, Te‐Te (3.00 Å), Ge‐Sb (2.80 Å) and Ge‐Ge (2.63 Å) pairs. In the case of the Ge‐Ge and Te‐Te pRDFs, the second maximum, respectively at 4.0   and 4.21 Å  is much more prominent than the first one at 2.63 Å and 3.00 Å. As regards the Ge‐Sb pRDF, the first peak is only slightly lower than the second one. Overall, the fraction of Ge‐Ge, Ge‐Sb, Sb‐Sb, and Te‐Te bonds is lower than in the liquid phase, whereas the fraction of Ge‐Te and Sb‐Te bonds increases upon quenching. Our results agree well with those of ref. [34]: there are a few minor discrepancies, such as the position of the second maximum of the Te‐Te pRDF (4.21 Å vs 4.16 Å ). We note that our Ge‐Te and Sb‐Te bond lengths are larger than those obtained from extended X‐ray absorption fine structure (EXAFS) experiments^[^ ^35^, ^36^ ^]^: this is a well‐known problem of PBE‐based simulations (or, according to ref. [37], of the use of a GTH pseudopotential for Ge), which the D3 corrections apparently do not cure. Interestingly, the Te‐Te pairs contribute significantly to the second peak of the RDF at a distance of approximately $\approx \;\sqrt{2}\;\bar{r}$, where $\bar{r}$ can be the Ge‐Te or Sb‐Te maximum. It is noteworthy that all the peaks of a‐GST‐225 sub‐ADFs occur at approximately 90°, as evidenced in **Figure** **8**. This observation suggests that the system is comprised of a significant number of Te‐Ge(Sb)‐Te components. Specifically, this arrangement entails a right angle bond between Te‐Ge(Sb)‐Te triplets, such that the diagonal distance between Te‐Te measures approximately $\approx \sqrt{2}\;\bar{r}$. The a‐GeTe structure shows similar properties: the first peak of the RDF consists predominantly of Ge‐Te bonds (peak at 2.77 Å) but also contains a fraction (lower than in liquid GeTe) of Ge‐Ge and Te‐Te bonds, whose peaks are centered at 2.61 and 2.94 Å,  respectively. The second peak is due to the Ge‐Ge and Te‐Te pairs, centered around 4 Å.

We compute the CNs following the protocol outlined in Section 3.1 and Equation (3) to establish the first coordination shell. In **Table** **6**, we list all the *r*_*cut*_ values used in our analysis of a‐GST‐225 and a‐GeTe.

**Table 6:** First maxima ($\bar{r}$) and first minima (*r*_*cut*_) of the pRDFs of a‐GST‐225 and a‐GeTe at *T* = 300 K. All the distances are expressed in Å  units.

	a‐GST‐225	a‐GeTe
	Conf	Conf
${\bar{r}}_{Ge-Ge}$	2.63	2.61
$r_{cut}^{Ge-Ge}$	2.9	3.2
${\bar{r}}_{Ge-Te}$	2.80	2.77
$r_{cut}^{Ge-Te}$	3.5	3.5
${\bar{r}}_{Te-Te}$	3.0	2.94
$r_{cut}^{Te-Te}$	3.25	3.2
${\bar{r}}_{Ge-Sb}$	2.8	/
$r_{cut}^{Ge-Sb}$	3.5	/
${\bar{r}}_{Sb-Sb}$	2.96	/
$r_{cut}^{Sb-Sb}$	3.5	/
${\bar{r}}_{Sb-Te}$	2.94	/
$r_{cut}^{Sb-Te}$	3.5	/

The average partial and total CNs from our analysis, as shown in **Table** **7**, differ from those reported in refs. [34, 38, 39] due to the use of different *r*_*cut*_ values. We observe that in a‐GST‐225 Ge primarily forms bonds with Te, and its coordination is primarily 4 and 5; Sb and Te have coordination peaked at 5 and 3, respectively (refer to Section S6.1, Supporting Information). Concerning a‐GeTe, the Ge coordination is peaked at 4 and 5, while the Te coordination is mostly 3 and 4 (Figure S19, Supporting Information). However, when employing the cutoff radii specified in refs. [34, 38], we obtain CNs (see Section S6.2, Supporting Information) that concur with those two works. As expected, the 8 − *N* rule is not fulfilled: the total CNs *N*_*Ge*_, *N*_*Sb*_ and *N*_*Te*_ in Table 7 are larger than 4, 3, and 2 in all cases considered. Excluding the Te atoms of the CM, Table 7 shows that a‐GST‐225 has Ge‐Ge, Sb‐Sb, and Ge‐Sb bonds with coordination numbers of 0.13, 0.79, and 0.52, respectively. The three CN values decrease and match those of refs. [34, 38, 39] when using the same *r*_*cut*_ parameters. The CNs of the X‐Te pairs increase moderately upon inclusion of the bonds with the Te atoms of the CM, indicating weak interaction between a‐GST‐225 and the CM even at 300 K. In a‐GeTe, the Ge‐Ge, Ge‐Te, and Te‐Te bonds have CNs of 1.00, 3.50, and 0.23, respectively, if the Te atoms of the CM are excluded. Inclusion of these atoms leads to significantly larger CNs, which reflects the stronger chemical interaction between the PCM and the CM at the interface.

**Table 7:** Average partial and total CNs of a‐GST‐225 and a‐GeTe at *T* = 300 K. The same notation of Table 4 is used.

a‐GST‐225 (300 K)									
	*N*_*Ge* − *Ge*_	*N*_*Ge* − *Sb*_	*N*_*Ge* − *Te*_	*N*_*Sb* − *Sb*_	*N*_*Sb* − *Te*_	*N*_*Te* − *Te*_	*N*_*Ge*_	*N*_*Sb*_	*N*_*Te*_
Conf	0.13	0.52	4.00	0.79	3.49	0.55	4.65	4.80	3.55
Conf+	0.13	0.52	4.12	0.79	3.68	0.59	4.77	4.99	3.71

a‐GeTe (300 K)									
Conf	1.00	/	3.5	/	/	0.23	4.50	/	3.73
Conf+	1.00	/	3.85	/	/	0.44	4.85	/	4.29

In Figure 8, we present the sub‐ADFs of a‐GST‐225. These sub‐ADFs reveal the presence of octahedral structures: there is a main peak at 90° along with a less pronounced peak at 170° degrees. The second peak is observed in all three sub‐ADFs, but it is more distinct in the case of Sb and Ge. Upon examining the pADFs resolved for various atomic triplets (Section S6.3, Supporting Information), it becomes apparent that the peak at 90° in the Ge sub‐ADF is attributed to the Te‐Ge‐Te triplets. On the other hand, the Sb‐Ge‐Te and Ge‐Ge‐Te triplets exhibit features at angles around 109.5° degrees, indicative of the presence of tetrahedral environments. The triplets involved in Te and Sb sub‐ADFs only show features at 90°.

Analogously, the a‐GeTe sub‐ADFs are compatible with octahedral structures and display a peak around 90° but the peak of the Ge sub‐ADF is shifted to slightly higher values. The pADFs support this observation: the peak of the Te‐Ge‐Te triplets is similar to the one of the Ge sub‐ADF, while Ge‐Ge‐Ge and Ge‐Ge‐Te show clear features at around 109.5° degrees. Concerning the pADF of the Ge‐Te‐Ge triplets, its behavior reflects the one of the Te sub‐ADF. Note that the long tail displayed by this pADF does not correspond to tetrahedrally coordinated Te, as further discussed below. The Te‐Te‐Te partial ADF of a‐GeTe exhibits a peak at 90° and another beyond 150°, similarly to the one of a‐GST‐225; however, their amplitudes are disproportionate, with the one at large angles related to the Te‐Te‐Te chains being much more prominent than the one centered around 90°. We have also calculated the Te‐Te‐Te ADFs including the second Te‐Te coordination shell (Section S6.3, Supporting Information) to compare with the model for a‐GeTe proposed in ref. [40], which consists of a disordered *fcc*‐type Te sub‐lattice interspersed with randomly arranged chains of Ge atoms in tetrahedral coordination. The ADFs are in fair agreement with the one of ref. [40] and the discrepancies can be ascribed to the presence of the interfaces, as reported in Section S6.3 (Supporting Information).

**Figure 9:**
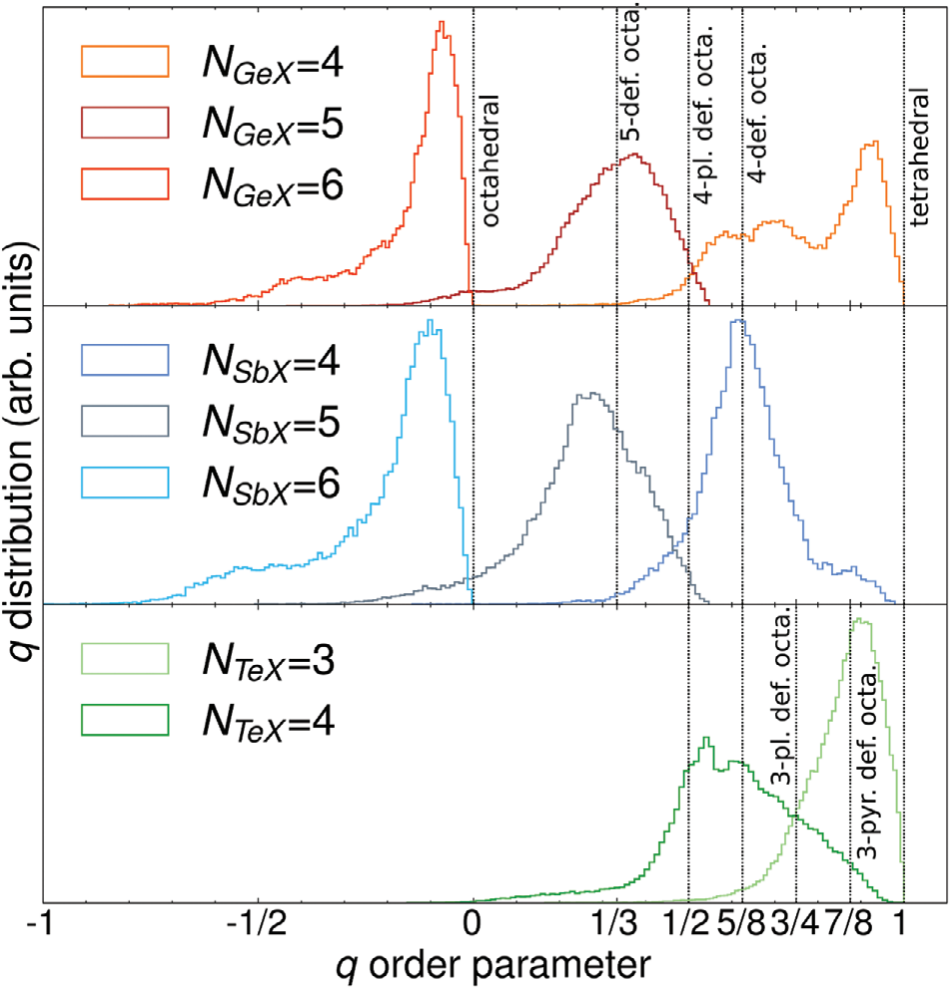
*q*‐distributions of a‐GST‐225 at *T* = 300 K resolved for different central atoms. Upper plot: four,five,sixfold Ge coordination. Mid plot: four,five,sixfold Sb coordination. Lower plot: three,fourfold Te coordinations. The black vertical lines indicate specific environments. All the distributions are normalized to one.

As discussed in Section 3.1, a more precise indicator of tetrahedral and octahedral geometry is given by the Errington‐De Benedetti parameter *q*. The Ge *q*‐distribution with *n* = 4 in a‐GST‐225 indicates the coexistence of tetrahedral and defective octahedral structures, as depicted in **Figure** **9**. Instead, the Ge atoms with *n* = 5 and *n* = 6 coordination have predominantly octahedral environment. In contrast, the Te and Sb *n* = 4 *q*‐distribution does not exhibit any signature of tetrahedral geometry, as supported by previous work,^[^ ^39^ ^]^ but shows a variety of defective octahedral structures. By integrating the tetrahedral peak of the *n* = 4 Ge *q*‐distribution in the range of 0.8 − 1.0 (as done in ref. [39]) we determine the fraction *p* of tetrahedral Ge atoms among the ones whose coordination is four. From this, we straightforwardly obtain a concentration of tetrahedral Ge in the system of 17.5%, which is a bit lower than the values obtained in the literature from pure PBE simulations, typically in the range 20–30%.^[^ ^39^, ^41^ ^]^ Upon closer analysis, it becomes evident that the vast majority (nearly 80%) of Ge atoms in tetrahedral environment form at least one bond with Ge or Sb atoms, as illustrated in Section S6.4 (Supporting Information). Specifically, among the nearest neighbors of the Ge atoms with tetrahedral coordination, 6.3% are Ge, 71% are Te, and 22.6% are Sb.

We show the a‐GeTe *q*‐distributions in Section S6.4 (Supporting Information): the concentration of tetrahedral Ge in GeTe is equal to 14.6% and most of the tetrahedral structures contain at least one Ge‐Ge bond. This concentration is also lower than the values typically reported in the literature for pure PBE simulations.^[^ ^42^ ^]^ These results suggest that the D3 corrections may lead to lower concentrations of tetrahedra with respect to PBE models. However, additional amorphous models should be generated to assess whether this is a genuine effect or simply a statistical fluctuation.

As done in Section 3.1, we calculate the ALTBC for the amorphous phases at 300 K to investigate the presence of Peierls‐like distortion. As evidenced by the *q*‐distributions and ADFs, there are numerous defective and distorted octahedral structures, and it is precisely within these structures that one would expect to find such distortions. The total ALTBC plots of a‐GeTe shows two well‐separated peaks (in line with refs. [43, 44]), indicative of Peierls‐like distortions. These distortions are mainly present in triplets of quasi‐aligned atoms with central Te atoms.

**Figure 10:**
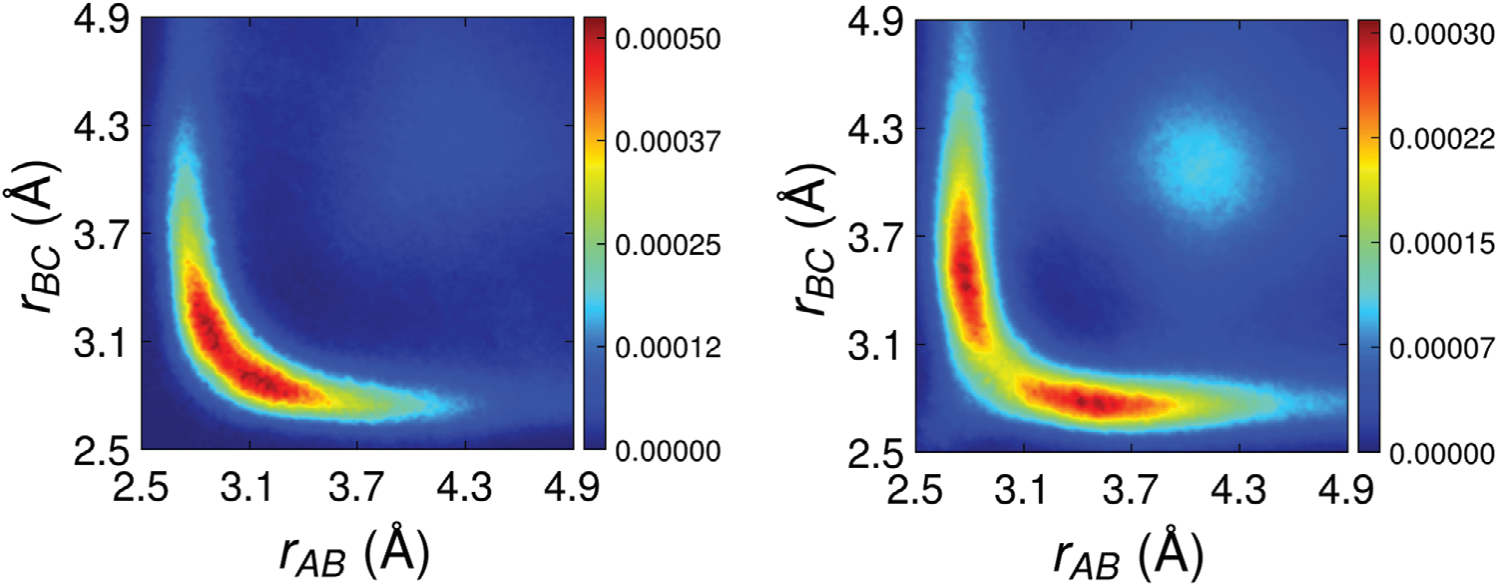
ALTBCs of the two a‐PCMs at *T* = 300 K. The left plot shows the octahedral Ge‐centered ALTBC of a‐GST‐225. The right plot refers to the total ALTBC of a‐GeTe.

The ALTBC of a‐GST‐225 exhibits an elongated peak centered on the diagonal. This is in agreement with previous work based on standard PBE functionals,^[^ ^45^ ^]^ whereas simulations based on the vdW density functional DF2^[^ ^46^ ^]^ show more sharply defined atomic connectivities, resulting in separate peaks even in the liquid phase.^[^ ^28^ ^]^ If we focus only on the environment of Ge atoms, we observe the signature of Peierls distortions in a‐GST‐225 as well (see **Figure** **10**) when considering octahedral Ge atoms.

### 4.2 Rings

**Figure 11:**
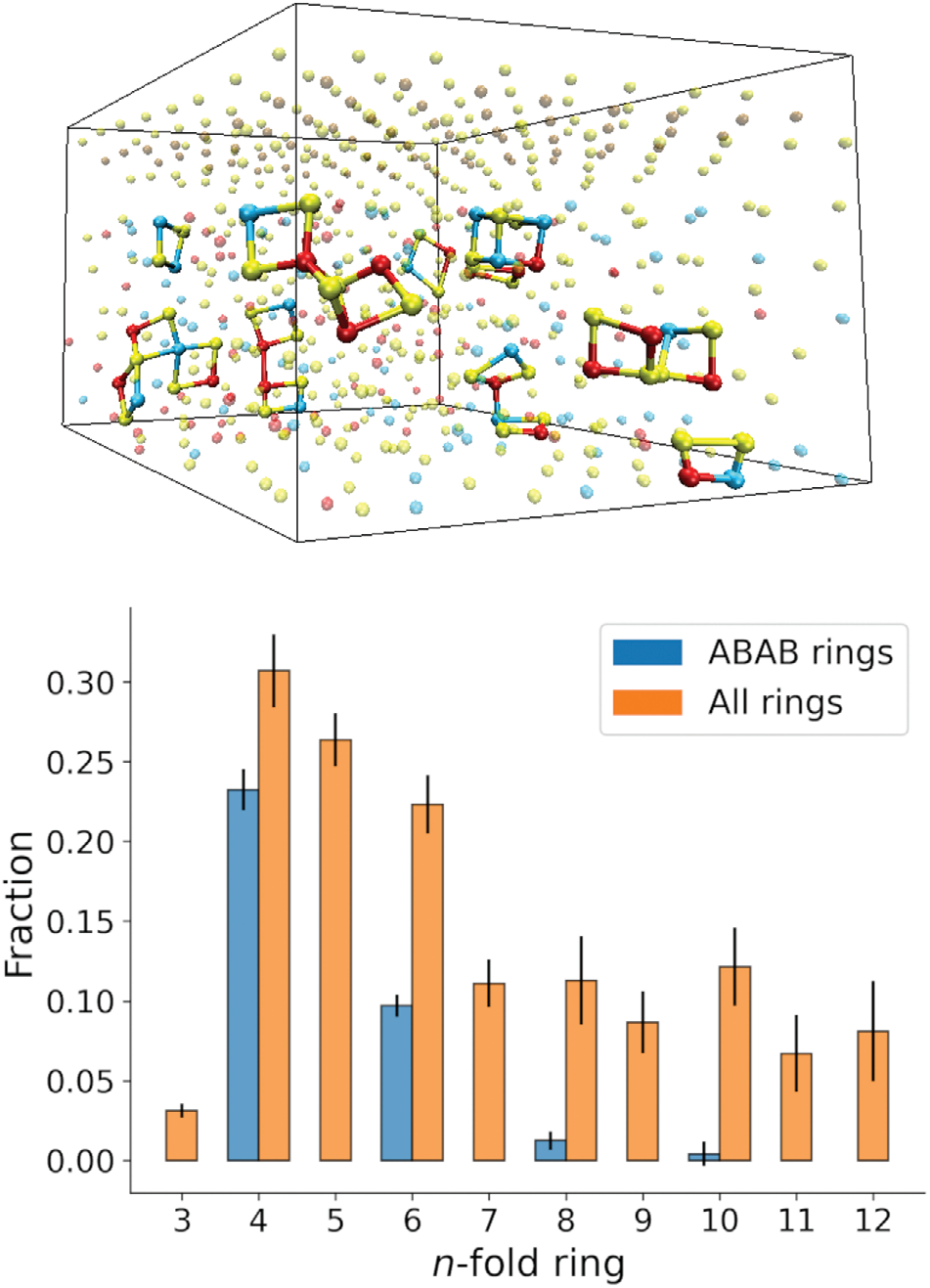
Distribution of primitive rings in a‐GST‐225 at *T* = 300 K. The upper plot shows a snapshot of the a‐GST‐225 heterostructure displaying some fourfold primitive rings. The vertical black bars in the ring histogram are error bars computed with the RINGS code.

We assess the intermediate‐range order through the ring statistics shown in **Figure** **11**. This method provides important insights into the crystallization kinetics of PCMs: more specifically, Akola and Jones^[^ ^34^ ^]^ and Lee and Elliott^[^ ^47^ ^]^ pointed out the significance of the ABAB squares, i.e., four‐membered rings with heteropolar bonds (A = Ge,Sb, and B = Te), as the building blocks not only of the rocksalt‐like crystalline phase of GST‐225 and GeTe, but also of their amorphous state. They also argued that fast crystallization from the amorphous state may stem from the rapid reorientation and ordering of these rings (which are randomly arranged in a‐GST‐225 and a‐GeTe) to form a rocksalt‐like structure.

We compute the ring statistics using an algorithm primarily based on the King‐Franzblau shortest path search,^[^ ^48^, ^49^ ^]^ which is now integrated into the RINGS code.^[^ ^50^ ^]^ The statistics is calculated up to rings of size *n* = 12, with the appropriate cutoffs deduced from the pRDFs. The distribution of primitive rings in a‐GST‐225 is shown in Figure 11, whereas the a‐GeTe distribution is included in Section S6.5 (Supporting Information). In agreement with previous work,^[^ ^34^, ^39^, ^47^ ^]^ we find a predominant number of four‐membered rings, showing that confinement does not influence the ring statistics compared to the bulk phase. We note, however, that GST‐225 layers confined in PCHs are expected to recrystallize into the hexagonal phase, similarly to Sb_2_Te_3_, otherwise the vdW gaps separating them from TiTe_2_ would be destroyed. Thus, the role played by the ABAB rings in the SET process of PCHs is not obvious and deserves further investigation.

## 5 Conclusion

In summary, we have investigated superlattice models of GST‐225/TiTe_2_ and GeTe/TiTe_2_ containing a single TiTe_2_ trilayer and ultrathin PCM slabs with eight or nine atomic layers. In the fully crystalline phase, the GST‐225/TiTe_2_ PCH is characterized by nearly perfect interfaces with weak chemical interactions between the atoms of the two blocks. In the case of GeTe/TiTe_2_ atomic corrugations are present at the interface and the chemical interaction is almost twice as strong. In spite of these differences, our AIMD simulations indicate that, by a controlled heating and quenching protocol, it is possible to melt down and amorphize the two confined PCMs without destroying the TiTe_2_ crystalline layer. In this respect, these superlattice models behave differently from the GST/Sb_2_Te_3_ models forming the so called interfacial PCMs,^[^ ^24^, ^51^ ^]^ in which the Sb_2_Te_3_ layers are not sufficiently robust (due to their low melting temperature) to serve as crystalline spacers between the actively switching GST layers.^[^ ^52^ ^]^ In our simulations, the use of a single TiTe_2_ trilayer was dictated by computational convenience. Using thicker CM layers may further improve the robustness of the PCH. It would also be interesting to investigate the ability of other layered dichalcogenides to behave as CMs. For instance, it was pointed out in ref. [53] that, among the transition metal tellurides, four of them (ZrTe_2_, MoTe_2_, RhTe_2_, and PtTe_2_) are potentially promising candidates, since they have both high melting temperatures (above 1400 K) and a trigonal or hexagonal structure with vdW‐like gaps, which is geometrically compatible with the Sb_2_Te_3_ (and, thus, the GST‐225) lattice.

Our simulations also show that, on timescales of hundreds of ps, a single trilayer of TiTe_2_ effectively prevents diffusion of the atoms of the PCM across it at temperatures of the order of 1300 K, at which the PCM is in the liquid state. We expect that thicker CM slabs should act as diffusion barriers even more effectively.

Notwithstanding these promising results, it must be obviously emphasized that in experiments many effects could in principle undermine the stability of the PCH, including the formation of defects during the growth of the PCH or during cycling (due, for instance, to the possible atomic diffusion across the CM on longer time scales than those simulated in our work and/or upon extensive cycling), as well as the melting of the confining layers owing to too intense electrical stimuli. Thus, both the growth conditions and the heating protocols must be carefully tuned and controlled to minimize these issues.

Our results also indicate an excess of Te atoms at the two interfaces in the PCM region for GeTe and, to a lesser extent, GST‐225. This is relevant to the SET process of the PCH. Indeed, it was shown by Acharya et al. in ref. [27] that, due probably to this excess, a thin GeTe slab in a superlattice recrystallizes into a disordered phase with Te layers at both interfaces and a high concentration of antisite defects and vacancies. Although in their work an artificial capping layer made by a frozen bilayer of crystalline GeTe was considered, instead of a TiTe_2_ layer (since a neural‐network potential capable of describing only Ge‐Ge, Ge‐Te, and Te‐Te interactions was employed), we believe that their findings should also hold for more realistic models of the GeTe/TiTe_2_ interface. Acharya et al. also found that the crystal growth velocity of nanoconfined GeTe is a factor of two lower than the bulk one at the temperature of maximal crystallization speed. This reduction does not represent an obstacle for phase‐change applications, since the crystal growth velocity remains very high. Nevertheless, the formation of a disordered GeTe phase in the SET process may have an impact on the performance of the devices, by affecting properties such as the electrical contrast and the cycling endurance.

As far as the GST/TiTe_2_ PCH is concerned, we conjecture that the excess of Te atoms at both interfaces could promote the formation of the rhombohedral GST phase upon fast crystallization. Therefore, contrary to GeTe/TiTe_2_, a more ordered phase could form in GST/TiTe_2_ PCHs with respect to conventional devices based on the bulk PCM. In conclusion, the GST/TiTe_2_ PCH appears to be a promising candidate for phase‐change devices possessing most of the beneficial properties shown by the Sb_2_Te_3_/TiTe_2_ PCH but with the additional advantage of a more stable amorphous state.

## Conflict of Interest

The authors declare no conflict of interest.

## Supporting information

Supporting Information

## Data Availability

The data that support the findings of this study are available from the corresponding author upon reasonable request.
