# Supplementary material for: Ab Initio Study of Novel Phase‐Change Heterostructures
Source: Adv Sci (Weinh). 2024 May 29;11(29):2402375. doi: 10.1002/advs.202402375 (PMC11304324; doi:10.1002/advs.202402375)
Supplement: Supplementary file 1 — Supporting Information [file ADVS-11-2402375-s001.pdf]

ADVANCED SCIENCE
Open Access

Supporting Information

for *Adv. Sci.*, DOI 10.1002/advs.202402375

Ab Initio Study of Novel Phase-Change Heterostructures

*Riccardo Piombo, Simone Ritarossi and Riccardo Mazzarello**

Supplementary Materials of "Ab-initio study of novel phase-change heterostructures"

*Riccardo Piombo Simone Ritarossi Riccardo Mazzarello**

Dipartimento di Fisica, Università di Roma "La Sapienza", 00185 Rome, Italy
Email address: riccardo.mazzarello@uniroma1.it

1 Cell Optimization

We optimize the PCHs' cells using the QUICKSTEP code included in the CP2K package [1]. We use a mixed Gaussian and plane-wave basis set to expand the Kohn-Sham orbitals (KSOs) and the charge density. Regarding the KSOs, we employ a Gaussian-type basis set of triple zeta (double zeta for Ti atoms) plus polarization quality. As for the charge density, we use a plane wave basis with an energy cutoff of 320 Ry. We model the valence configurations of Ge, Sb, Te, and Ti ($4s^2\,4p^2$, $5s^2\,5p^3$, $5s^2\,5p^4$, and $4s^2\,3d^2$, respectively) using scalar-relativistic Goedecker-Teter-Hutter pseudopotentials [2]. We also incorporate the Van der Waals (VdW) correction DFT-Grimme D3 [3] including the calculation of the C_9 coefficient, representing a 3-body interaction term. We minimize the cells through a BFGS minimizer that relies on the diagonalization of the entire Hessian matrix. We compute the stress tensor at each step. We set strict convergence criteria: the maximum geometry change (MAX DR) is 10^{-5} Bohr, the maximum root mean square (RMS DR) geometry change is $5 \cdot 10^{-6}$ Bohr, the maximum force of each geometrical configuration (MAX FORCE) is $1.5 \cdot 10^{-6}$ Hartree/Bohr and finally the maximum root mean square force (RMS FORCE) of each geometrical configuration is 10^{-6} Hartree/Bohr. Finally, we ensure that the optimization process preserves the cells' symmetry. In **Table S1**, we compare the relaxed lattice parameters of bulk GeTe, GST-225 and $TiTe_2$ at T = 0 K obtained with pure PBE, PBE with D2 corrections [4] and PBE with D3 corrections.

	GST-225			GeTe			$TiTe_2$		
	no corr	D2	D3	no corr	D2	D3	no corr	D2	D3
a	4.32	4.20	4.35	4.24	4.19	4.24	3.81	3.79	3.79
c	17.50	17.19	17.05	10.94	10.46	11.03	6.74	6.58	6.56

Table S1: Comparison of a and c parameters of hexagonal bulk models of GST-225, GeTe, and $TiTe_2$ at T = 0 K. For GeTe the conventional hexagonal cell with 6 atoms is used. The wording "no corr" refers to the case of pure PBE calculations, while the others refer to D2 and D3 corrections added to the PBE functional. All the parameters are expressed in Å units.

2 Ab-Initio Molecular Dynamics Simulations

To determine the structural and dynamic characteristics of the confined PCMs in their crystalline (600 K), liquid (1300 K), and amorphous (300 K after quenching) phases we conduct ab initio molecular dynamics (AIMD) simulations using the CP2K package. In this context, we expand the charge density on a larger basis than the cell optimization setting, raising the energy cutoff to 500 Ry. The program employs periodic boundary conditions, with the Brillouin zone only sampled at the Γ point.

We use the Born-Oppenheimer molecular dynamics (BOMD) and the second-generation Car-Parrinello molecular dynamics (SGCPMD) to ensure efficiency and accuracy. The latter is a technique developed by Kühne et al. [5], which is faster than BOMD and standard CPMD but intrinsically dissipative. The dissipation is described by the intrinsic damping coefficient "noisy-gamma" γ_D, which depends on the system under consideration. γ_D is extracted from preliminary simulations. A second, extrinsic damping coefficient, γ_L, can also be added. After careful analysis, we set $\gamma_L = 4 \cdot 10^{-3} fs^{-1}$ and $\gamma_D = 3.5 \cdot 10^{-5} fs^{-1}$ for both PCH models.

We perform BOMD simulations with the Bussi thermostat [6] for warming up the system (first to 600 K, then to 1700 K). The Bussi thermostat allows for sampling the canonical ensemble through a stochastic

rescaling of the velocities. In our simulations, the rescaling occurs every 10 timesteps (5 fs). This approach ensures a rapid temperature increase, while also generating optimized wavefunctions at the appropriate temperature. These wavefunctions are subsequently employed to initiate the SGCPMD. The timesteps in our simulations are 0.5 fs for BOMD-Bussi and 2 fs for SGCPMD.

We generate the crystalline phases at 600K through the following protocol: initially we conduct BOMD-Bussi simulations to warm up the crystalline PCHs from 0 K to 600 K covering trajectories that lasted 1 picosecond. After that, we equilibrate the systems at 600K for 20 picoseconds using SGCPMD. Finally, we generate trajectories spanning 30 picoseconds with SGCPMD, during which we calculate and analyze various observables.

We implement the two methods mentioned above to simulate also the PCH partial liquid phase at 1300K with some variations. When implementing the BOMD-Bussi simulation, we increase the temperature from 600 K to 1700 K, encompassing a trajectory that lasted 1 picosecond. Next, we apply the SGCPMD method holding the system at 1700 K for 10 picoseconds to accelerate the melting of the PCMs while ensuring that the CM retains its crystalline structure throughout the simulation. Following this, we reduce the temperature to 1300 K and equilibrate the system for 30 picoseconds. Finally, we generate a trajectory spanning 50 picoseconds, during which we compute and analyze all relevant observables.

To generate the amorphous phases, we employ SGCPMD only. We conduct an initial quenching with a rate of $10^{15}K/s$ to bring the system's temperature from 1300K to 900K. After equilibrating the system at 900K for a few picoseconds, we perform a second quenching with a rate of $10^{13}K/s$ until reaching a temperature of 300K. Then we generate trajectories of approximately 12 picoseconds and calculate and analyze the relevant observables (excluding the initial 2 ps for the analysis).

3 Analysis of the VdW gaps

Prior to cell optimization, the two PCHs exhibit a layered structure in their crystalline phase, with the total atomic density profile $\rho(z)$ appearing as a sequence of Dirac delta functions, each with an amplitude corresponding to the number of atoms present in the layer. The thickness of the VdW gap in each PCH is determined by the peak-to-peak distance between the $\rho(z)$ components relative to the planes of the PCM and the CM facing each other. Thus, in the GST-225/$TiTe_2$ heterostructure, the gap thickness is defined by the distance between the Te planes of both GST-225 and $TiTe_2$ facing each other. For GeTe/$TiTe_2$, it is calculated from the distance between the Te planes of $TiTe_2$ and the adjacent Ge or Te plane of GeTe.

However, the system's symmetry is reduced after cell optimization, and the layered structure may become distorted. This effect is more prominent at higher temperatures, as thermal motion further disrupts the crystalline order. Obviously, this effect reaches its maximum after partial melting of the PCM (as well as in the partly amorphous models at lower temperature). To calculate the VdW gap thickness in such instances, we determine the average position of the PCM and CM interfaces by identifying the local maxima of the $\rho(z)$ histogram. The VdW gap is then defined as the spatial region, depicted in cyan color, between the PCM and CM $\rho(z)$ nearest maxima, which are marked by dashed vertical lines in the bottom plots of **Figures (S1)** and **(S2)**. We use a Gaussian filter to increase the precision of the estimate of the peak values.

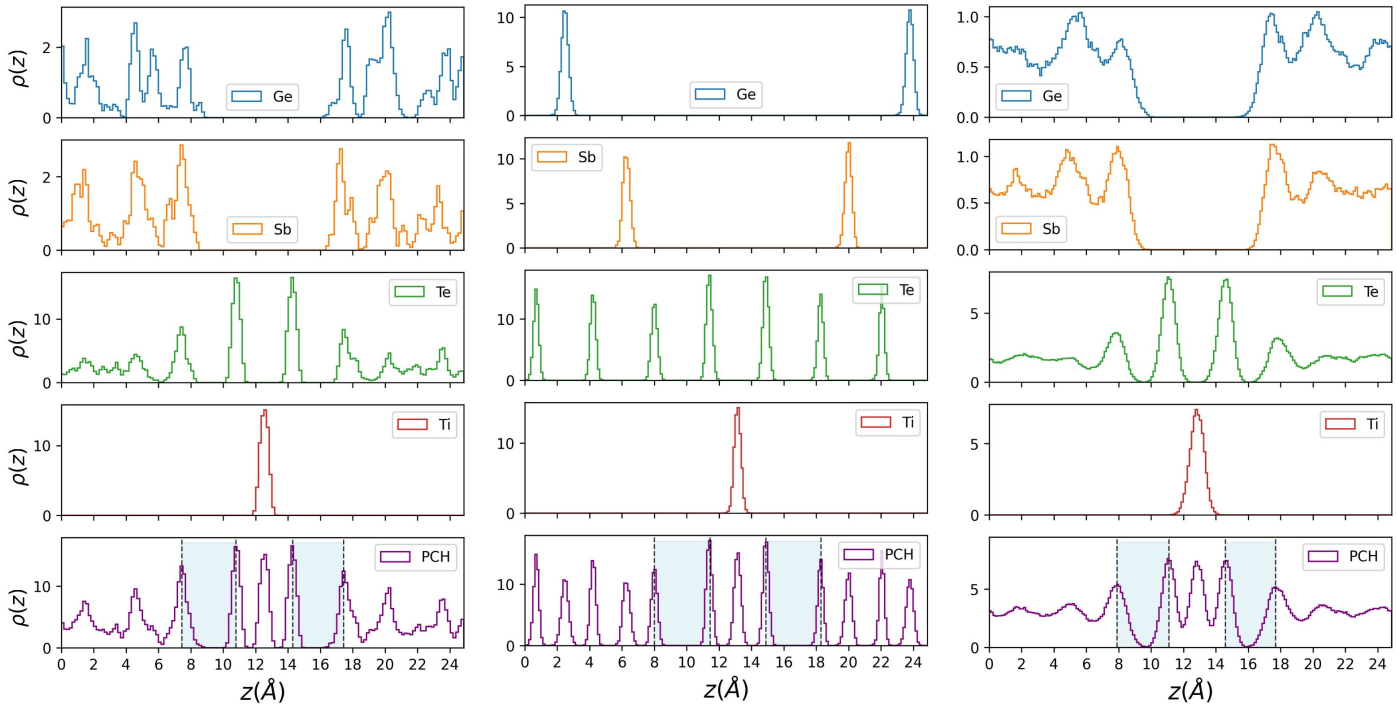

Figure S1: Atomic density profile $\rho(z)$ of the GST-225/$TiTe_2$ heterostructure. From left to right the $T = 300$ (amorphous), 600 and 1300 K cases are depicted. In each plot, from top to bottom, the Ge, Sb, Te, Ti and total atomic density profiles are shown. The black dashed lines in the bottom plots (PCH) depict the $\rho(z)$ maxima defining the thickness of the VdW gaps. The VdW gaps are the region of space shaded in cyan and enclosed between two $\rho(z)$ maxima.

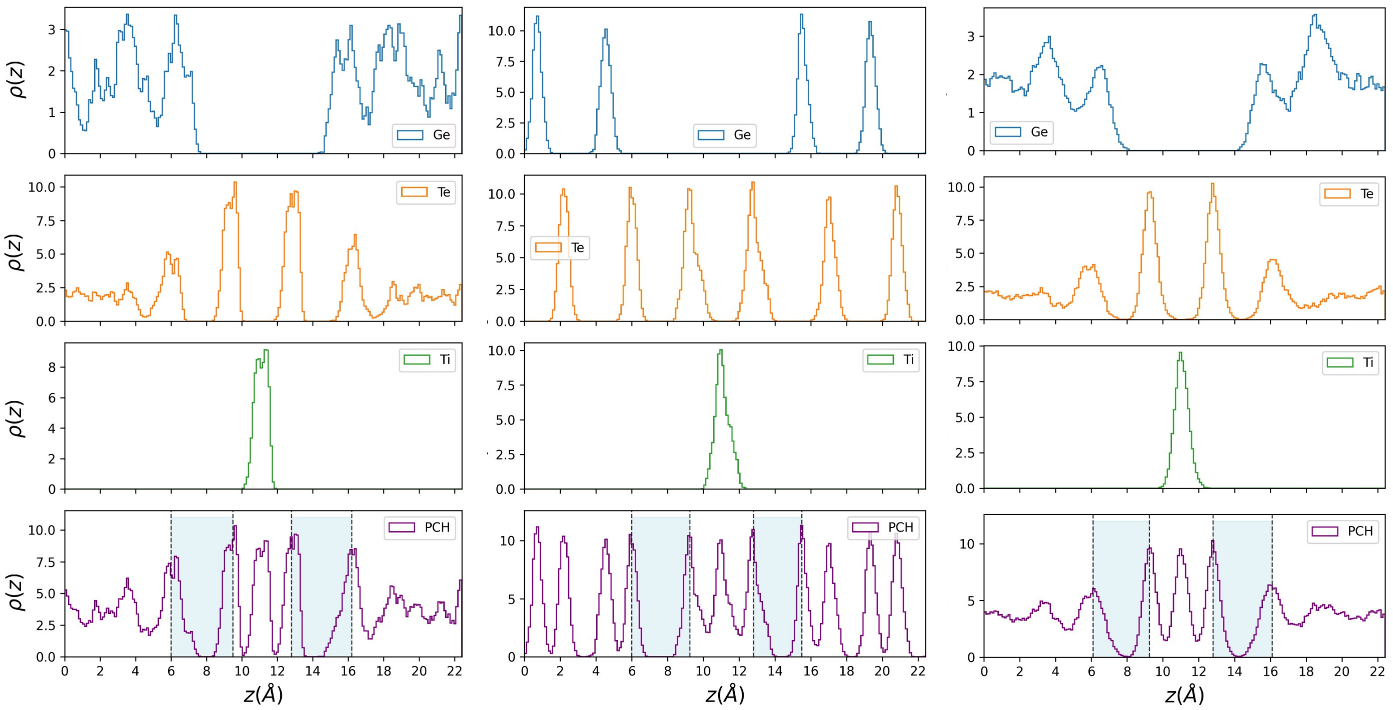

Figure S2: Atomic density profile $\rho(z)$ of the GeTe-225/$TiTe_2$ heterostructure. From left to right the $T = 300$ (amorphous), 600 and 1300 K cases are depicted. In each plot, from top to bottom, the Ge, Te, Ti and total atomic density profiles are shown. The black dashed lines in the bottom plots (PCH) depict the $\rho(z)$ maxima defining the thickness of the VdW gaps. The VdW gaps are the region of space shaded in cyan and enclosed between two $\rho(z)$ maxima.

4 Partial Melting

4.1 Coordination Numbers

The coordination numbers (CNs) are a useful metric for studying the spatial correlations between the individual particles of a system. They are particularly helpful for characterizing the structure around an atom or molecule. Liquids and amorphous systems can be described as complex bond networks with well-defined local structures (coordination shells). However, characterizing these coordination shells in terms of a fixed set of distances between particles is ineffective, as in a liquid at finite temperature, the individual atoms are in constant motion, and distances constantly fluctuate. Additionally, quenched amorphous systems suffer from statistical variability of the coordination pattern.

A more effective approach to characterizing disordered structures is using a distribution function of distances in the coordination shell to analyze the average structure near the particles of the system. This distribution function, called radial distribution function (RDF), measures the probability of finding two particles a distance r apart and displays peaks at particular r values, which are characteristic of the structure, with peak widths primarily determined by the temperature and density of the system. To calculate the CNs, we first determine the RDF using AIMD simulations and then identify its first peak. We then integrate the RDF up to the position r_{cut} of the first minimum to determine the number of atoms enclosed in a sphere of radius r_{cut} centered on each particle (see Eq.3 in the main text). This protocol is also valid for partial RDFs. It follows that CNs depends sensitively on the choice of r_{cut} and even a small variation can lead to significant changes in the CNs. This is why the RDF remains the primary benchmark for comparing our results with those indicated in [7, 8]. In fact, in the main text, we make it clear how many of our results align with those of these studies, despite having different CNs due to the use of different r_{cut}. In this section, we present the CNs at $T = 1300$ K that we would obtain if we used the r_{cut} values defined in the referenced works. As specified in **Tables S2** and **S3**, the third and fourth columns report CN values that are quite compatible. Furthermore, we report in **Figures(S3)** and **(S4)** the CNs $N_{\alpha\beta}$ as a function of r_{cut} of confined liquid GST-225 (l-GST-225) and confined liquid GeTe (l-GeTe) at $T = 1300$ K, where α and β denote atomic species.

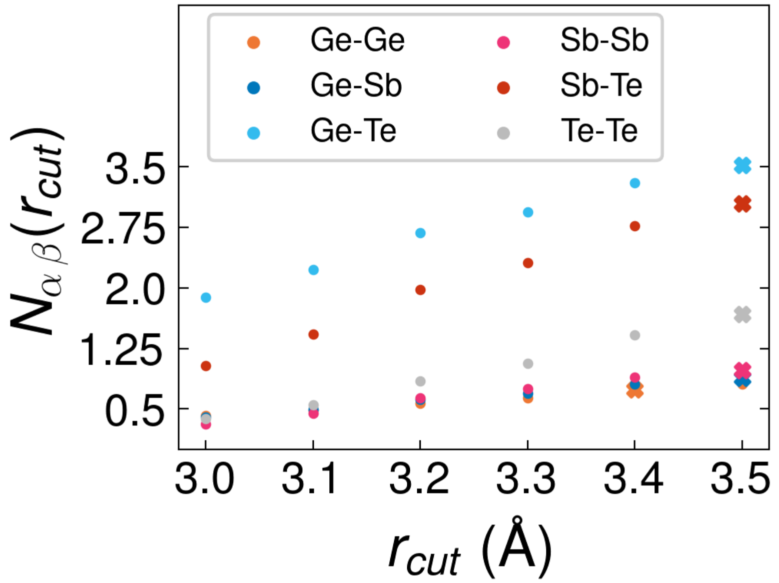

Figure S3: CN $N_{\alpha\beta}$ of confined l-GST-225 as a function of r_{cut} at $T = 1300$ K. Different colors refer to different $\alpha\beta$ atomic pairs. Crosses represent the CN values that we obtained by means of the cutoff radii specified in Table 4 of the main text.

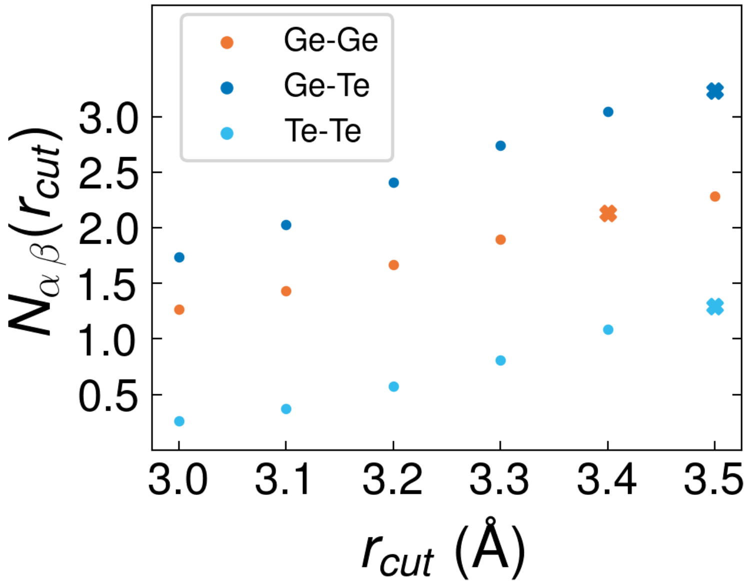

Figure S4: CN $N_{\alpha\beta}$ of confined l-GeTe as a function of r_{cut} at $T = 1300$ K. Different colors refer to different $\alpha\beta$ atomic pairs. Crosses represent the CN values that we obtained by means of the cutoff radii specified in Table 4 of the main text.

Pair	r_{cut} (Å) Ref. [7]	$N_{\alpha\beta}$ Ref. [7]	$N_{\alpha\beta}$
Ge-Ge	3.19	1.44	1.67
Ge-Te	3.36	2.80	2.85
Te-Te	3.40	0.69	1.09

Table S2: Comparison of CNs concerning confined l-GeTe. Column 2 outlines the cutoff radii utilized in the study by Weber et al. [7], while column 3 presents the overall CNs reported by the same authors in their work. In column 4, we present the total CNs we obtained when applying the cutoffs specified in column 2.

Pair	r_{cut} (Å) Ref. [8]	$N_{\alpha\beta}$ Ref. [8]	$N_{\alpha\beta}$
Ge-Ge	3.13	0.44	0.51
Ge-Sb	3.35	0.67	0.73
Ge-Te	3.50	3.38	3.53
Sb-Sb	3.50	0.84	1.00
Sb-Te	3.50	2.82	3.04
Te-Te	3.50	1.30	1.67

Table S3: Comparison of CNs concerning confined l-GST-225. Column 2 outlines the cutoff radii utilized in the study by Schumacher et al. [8], while column 3 presents the overall CNs reported by the same authors in their work. In column 4, we present the total CNs we obtained when applying the cutoffs specified in column 2.

4.2 Partial Angular Distribution Functions

Partial angle distribution functions (pADFs) serve as pivotal tools for characterizing the angular relationships among atomic triplets. Specifically, pADFs capture the statistical distribution of angles formed by atoms of species α, β and γ. The pADF quantifies the likelihood of observing a particular angle θ between the specified $\alpha\beta\gamma$ atomic triplet. This statistical metric enables a nuanced exploration of angular distributions, revealing insights into the local geometric arrangements and correlations inherent to the atomic structure. To compute the pADF we analyzed the trajectories obtained from AIMD simulations. We report in **Figures (S5)** and **(S6)** the pADFs relative to confined l-GST-225 and confined l-GeTe at $T = 1300$ K.

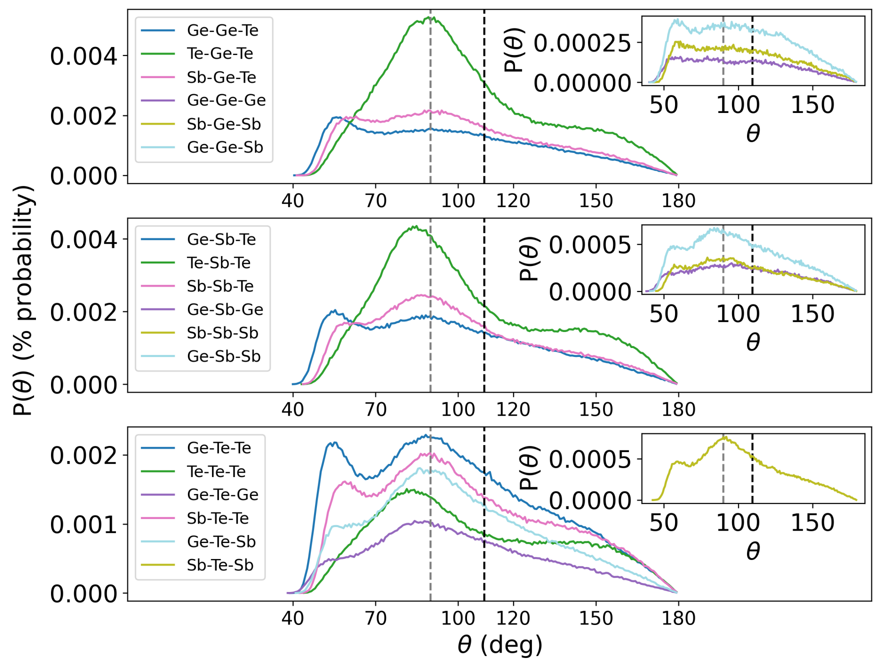

Figure S5: pADFs of confined *l*-GST-225 at T = 1300 K. The upper plot refer to all the Ge-centered triplets; the mid plot refer to all the Sb-centered triplets; the lower plot shows all the Te-centered triplets. Each pADF is normalized to the relative number of atomic triplets. The black dashed line is centered at 90° while the grey dash line is centered at 109.5°.

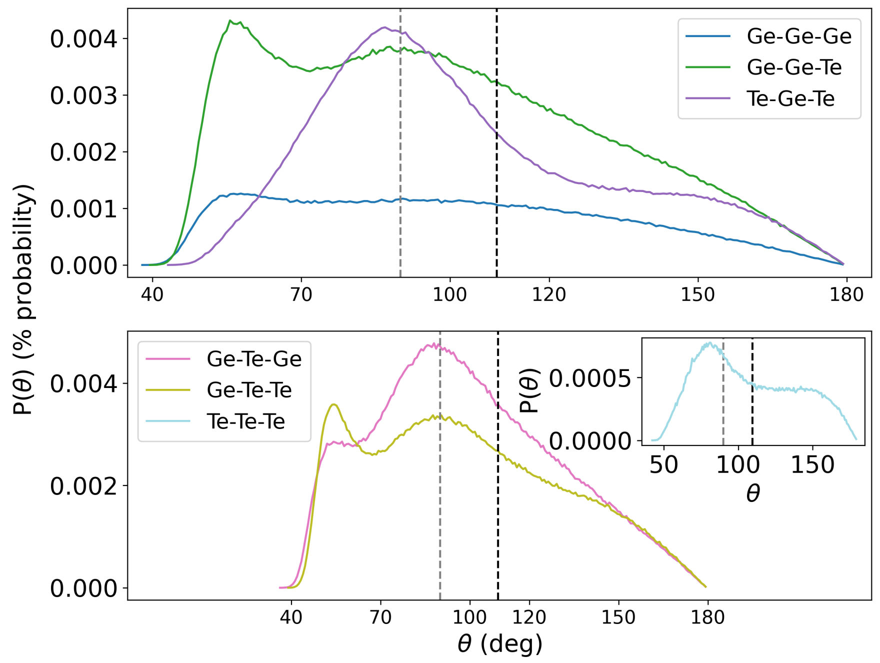

Figure S6: pADFs of confined *l*-GeTe at T = 1300 K. The upper plot shows all the Ge-centered triplets; the lower plot shows all the Te-centered triplets. Each pADF is normalized to the relative number of atomic triplets. The black dashed line is centered at 90° while the grey dash line is centered at 109.5°.

4.3 Sub-Angular Limited Three-body Correlation Functions

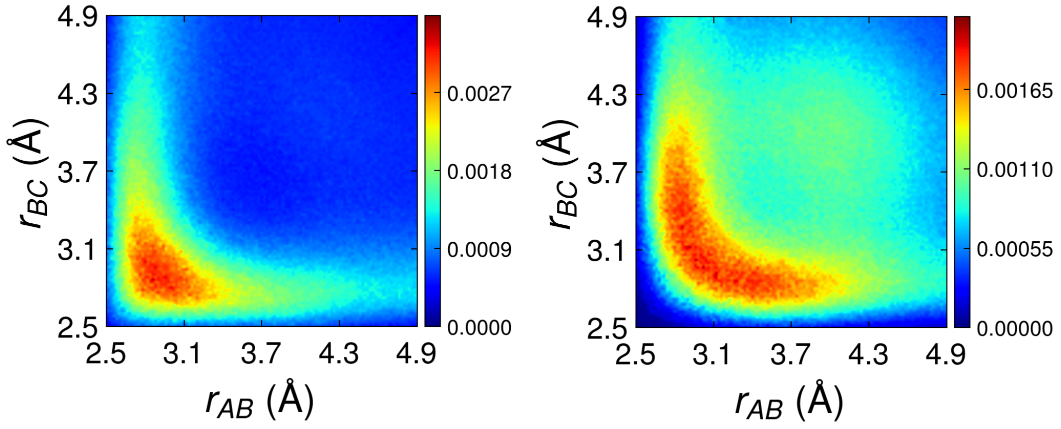

Figure S7: Sub-ALTBCs of confined *l*-GeTe at $T = 1300$ K. The left plot shows the Ge-centered ALTBC and the right plot shows the Te-centered ALTBC.

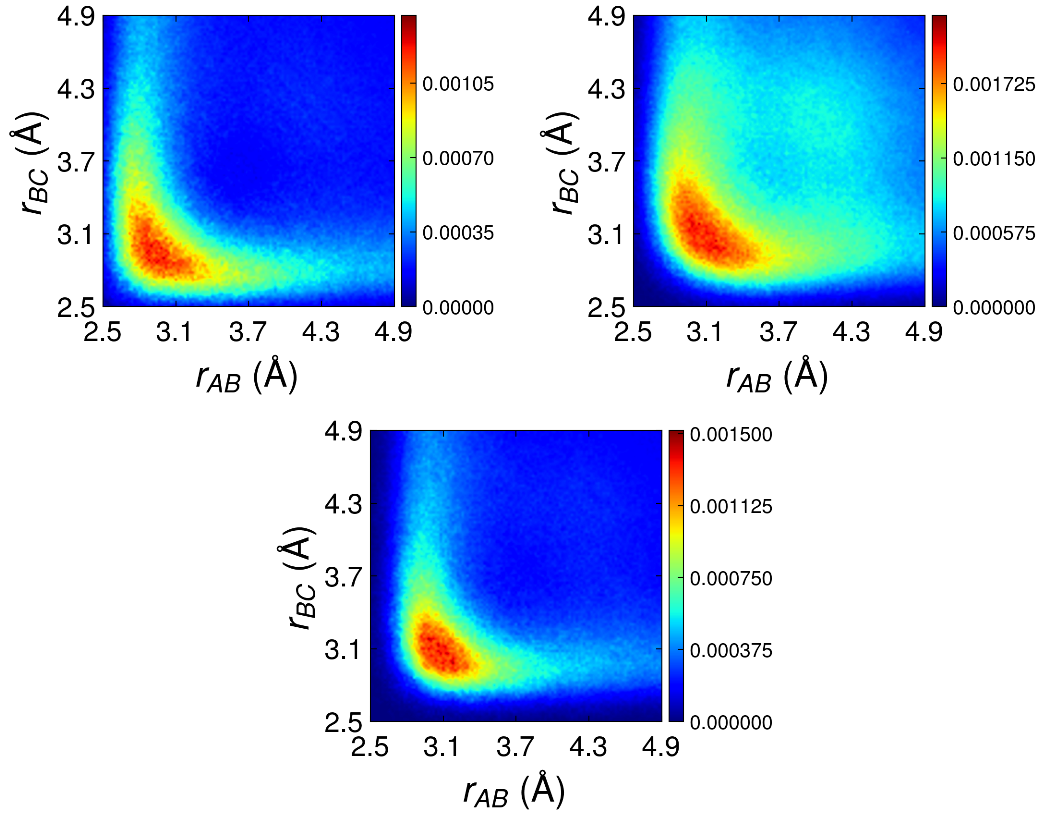

Figure S8: Sub-ALTBCs of confined *l*-GST-225 at $T = 1300$ K. The upper plots shows the Ge-centered (left) and Te-centered (right) ALTBCs. The lower plot shows the Sb-centered ALTBC.

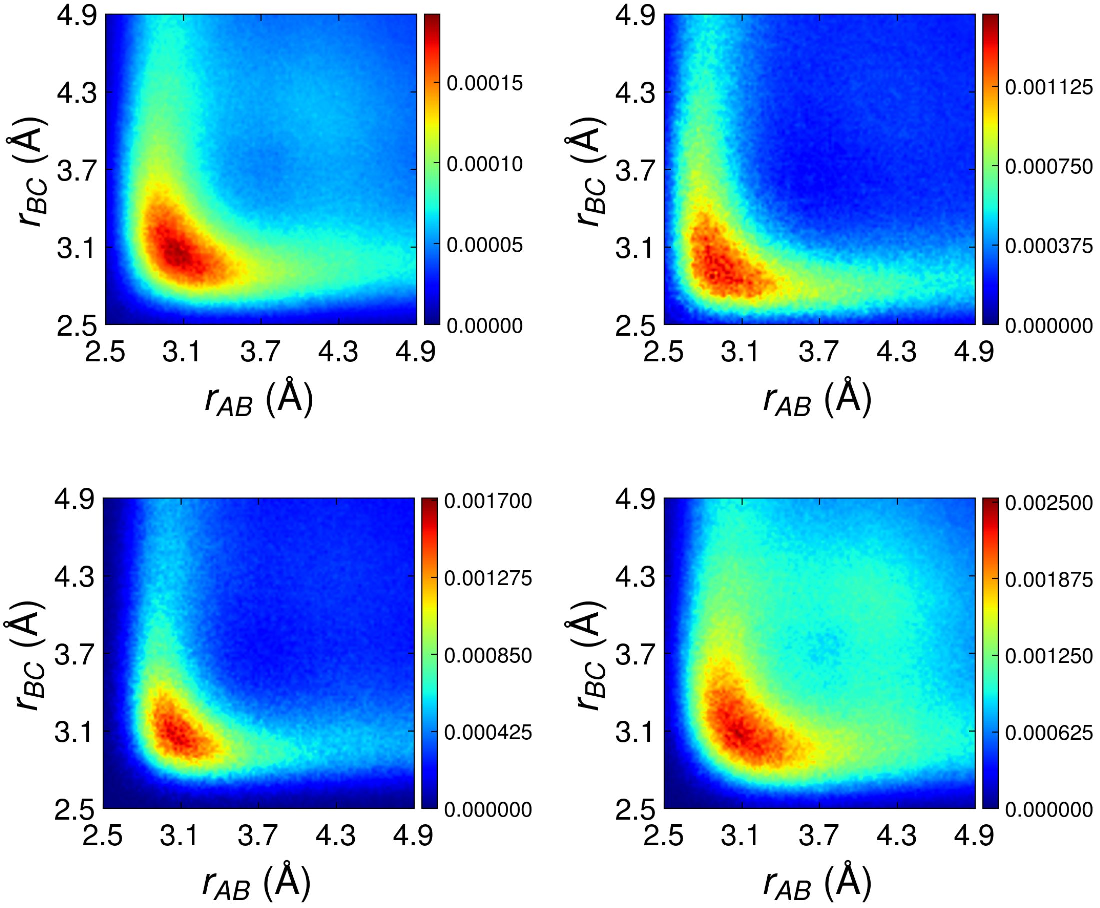

Figure S9: Total and sub-ALTBCs of bulk l-GST-225 at $T = 1300$ K. The upper plots show the total (left) and Ge-centered ALTBCs. The lower plots show the Sb-centered (left) and Te-centered (right) ALTBCs.

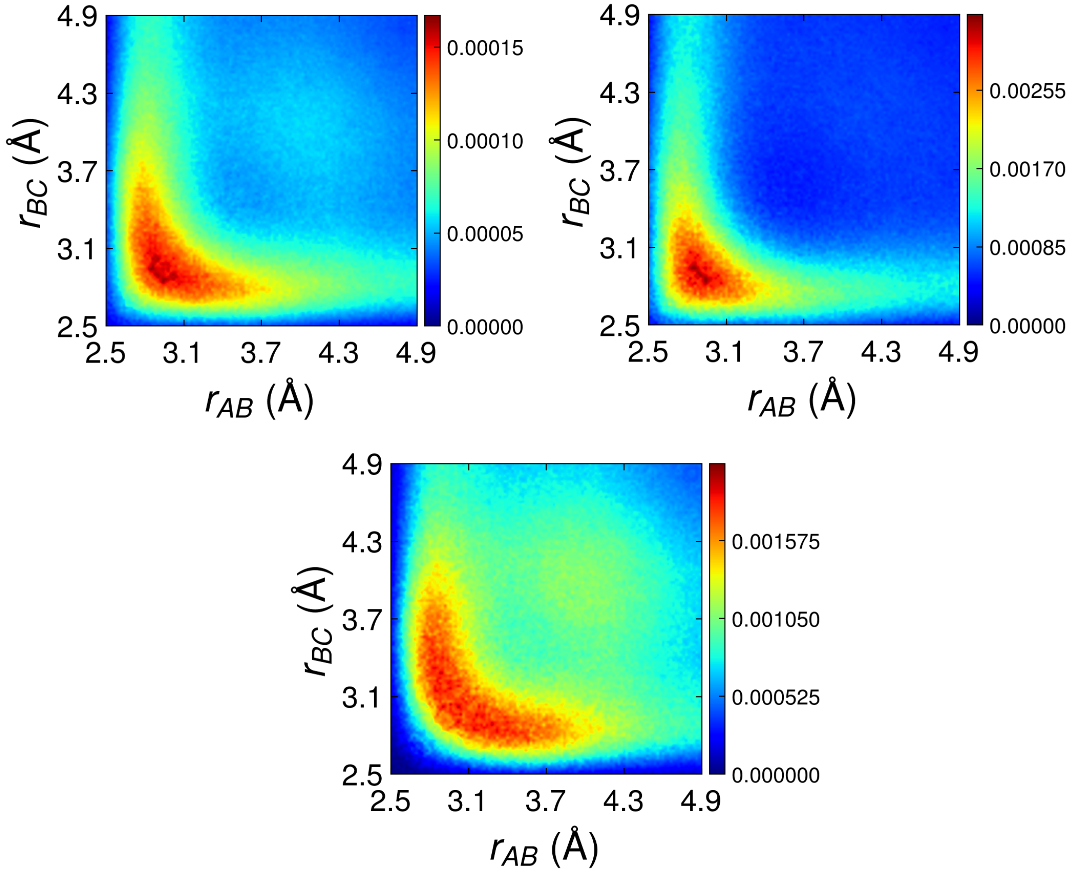

Figure S10: Total and sub-ALTBCs of bulk l-GeTe at $T = 1300$ K. The upper left plot shows the total ALTBC and the upper right plot shows the Ge-centered ALTBC. The lower plot shows the Te-centered ALTBC.

4.4 Coordination and q Order Parameter

The Errington-DeBenedetti q order parameter is a crucial metric in the study of phase transitions and structural properties of fluids. We report in **Figures(S11)** and **(S12)** the coordination of confined l-GST-225 and confined l-GeTe. We also report in **Figures(S13)** and **(S14)** the q-distributions relative to the prominent environments selected by the coordination in the previous two figures.

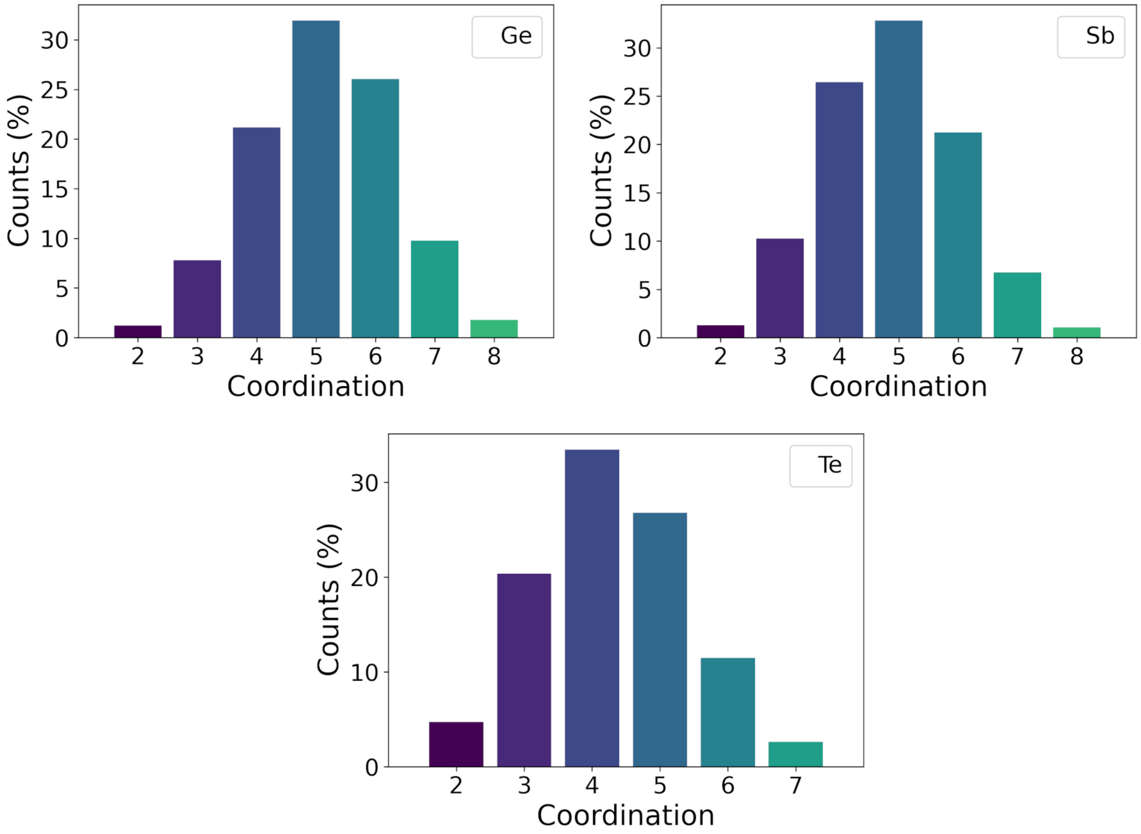

Figure S11: Coordination of confined l-GST-225 at $T = 1300$ K. The upper plots show Ge (left) and Sb (right) coordination; The lower plot shows Te coordination.

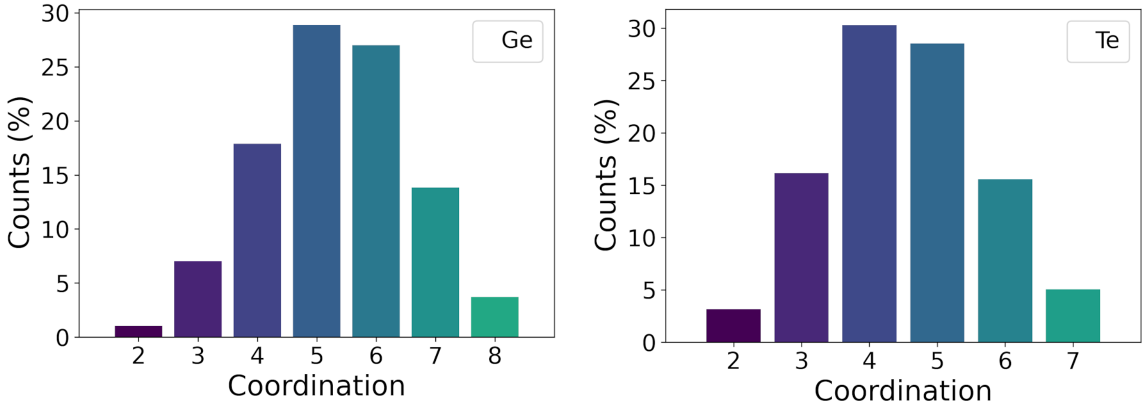

Figure S12: Coordination of confined l-GeTe at $T = 1300$ K. The left plot shows Ge coordination; the right plot shows Te coordination.

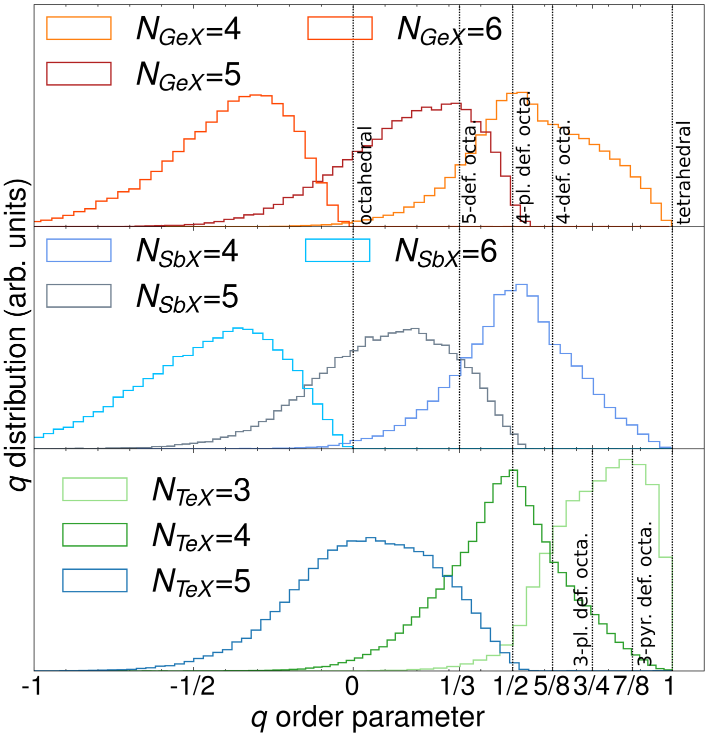

Figure S13: Errington-De Benedetti local order parameter q of confined l-GST-225 at $T = 1300$ K. The upper plot shows the 4, 5 and 6-fold coordination of Ge atoms; the mid plot shows the 4, 5 and 6-fold coordination of Sb atoms. The lower plot shows the 3, 4, and 5-fold coordination of Te atoms.

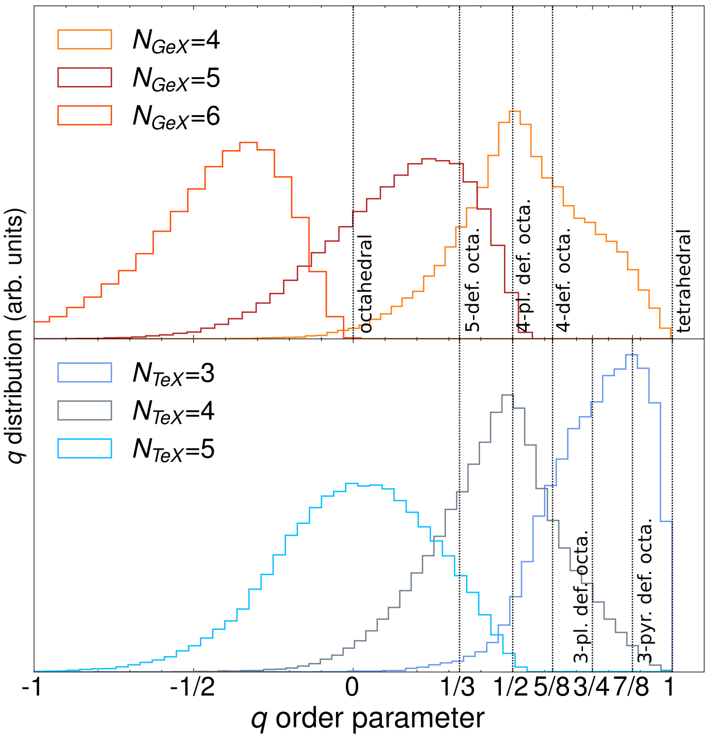

Figure S14: Errington-De Benedetti local order parameter q of confined l-GeTe at $T = 1300$ K. The upper plot shows the 4, 5 and 6-fold coordination of Ge atoms; the lower plot shows the 3, 4, and 5-fold coordination of Te atoms.

5 Diffusion Coefficient

The Einstein formula let us compute the diffusion coefficient D_z along the vertical (z) direction:

$$D_z = \frac{1}{2} \lim_{t\to\infty} \frac{d}{dt} \Big\langle \mathrm{MSD}_z(t) \Big\rangle. \tag{1}$$

The average in Eq.(1) designates an average over time and an average over several AIMD trajectories. To calculate $\mathrm{MSD}_z(t)$ from a trajectory, it is essential to divide such trajectory into several displacements. To that aim, we compute $\mathrm{MSD}_z(t)$ at equally spaced time points Δt, $2\Delta t$, $\cdots$, $T_{run}\Delta t$, where Δt represents the time step and T_{run} designates the length of the entire AIMD simulation. In this way, we derive $T_{run}(T_{run}-1)/2$ non-trivial forward displacements $z(j)-z(i)$ that correspond to time lags $(j-i)\Delta t$, where $1 \le i < j \le T$. The quantity $z(i)$ denotes the z atomic coordinate at time i. While this definition involves many distinct forward displacements at small time lags, it entails very few such displacements at large time lags. However, since the time step is fixed, we can disregard this and refer to the time lag t when selecting $j = (i+t)$. As a result, we align the definition of time lag with the time index and obtain the following formula for MSD_z, which can be applied to each trajectory:

$$\mathrm{MSD}_z(t) = \frac{1}{T_{run}-t} \sum_{i=0}^{T_{run}-t} [z(i+t) - z(i)]^2. \tag{2}$$

To compute Eq.(2), we wrote a Python code designed to handle different materials (GST-225, GeTe) with specific simulation parameters. The main functionalities include loading trajectory coordinates, dividing the simulation into sub-runs, determining the slab position for each atom, and calculating the time-lag MSD_z for each slab.
We begin by assigning each atom to a slab at time $t = 0$, relying on its z coordinate. Second, for all the atoms in a slab, we calculate the average in Eq.(1) in the interval $[0; t_{surv}]$ where the surviving time is defined in the main text. This operation is repeated every 1 ps (500 MD steps). Therefore, the second time window is $[1, t_{surv}+1]$ and so on until the last, N_w-th one, which is $[N_w, t_{surv}+N_w]$, where $t_{surv}+N_w$ is shorter but very close to the total duration of the simulation.
The trajectories of atoms within each slab during a specific sub-run are stored in a trajectory matrix organized such that each row corresponds to a trajectory of a single particle, and each column corresponds to a time step within the specified sub-run. After the trajectories are organized in matrices, Eq.(2) is calculated for each trajectory at each time lag over all pairs of time steps within the sub-run. This process is parallelized using multiprocessing to enhance computational efficiency. Then we perform the ensemble average marginalizing over the rows of the trajectory matrix. The result is a time-ensemble-average MSD_z of a sub-run of a specific slab. Repeating these process for each sub-run gives the set of red curves depicted in **Figures(S15)** and **(S16)**. From the average of these curves we obtain the black curves which are then fitted with a linear function in the region relative to diffusive regime. The result of the fit are listed in **Table S4** and refers to Fig.(6) of the main text.

Slab	$D_z \times 10^{-10}$ m/s^2	
	GST-225	GeTe
1	17.70	21.13
2	28.75	29.63
3	34.20	34.25
4	33.21	28.18
5	26.39	20.22
6	18.26	/

Table S4: D_z profile of the two confined l-PCMs at $T = 1300$ K as the slab number written in the first column varies. The region ascribed to confined l-GST-225 (l-GeTe) is divided into six (five) slabs.

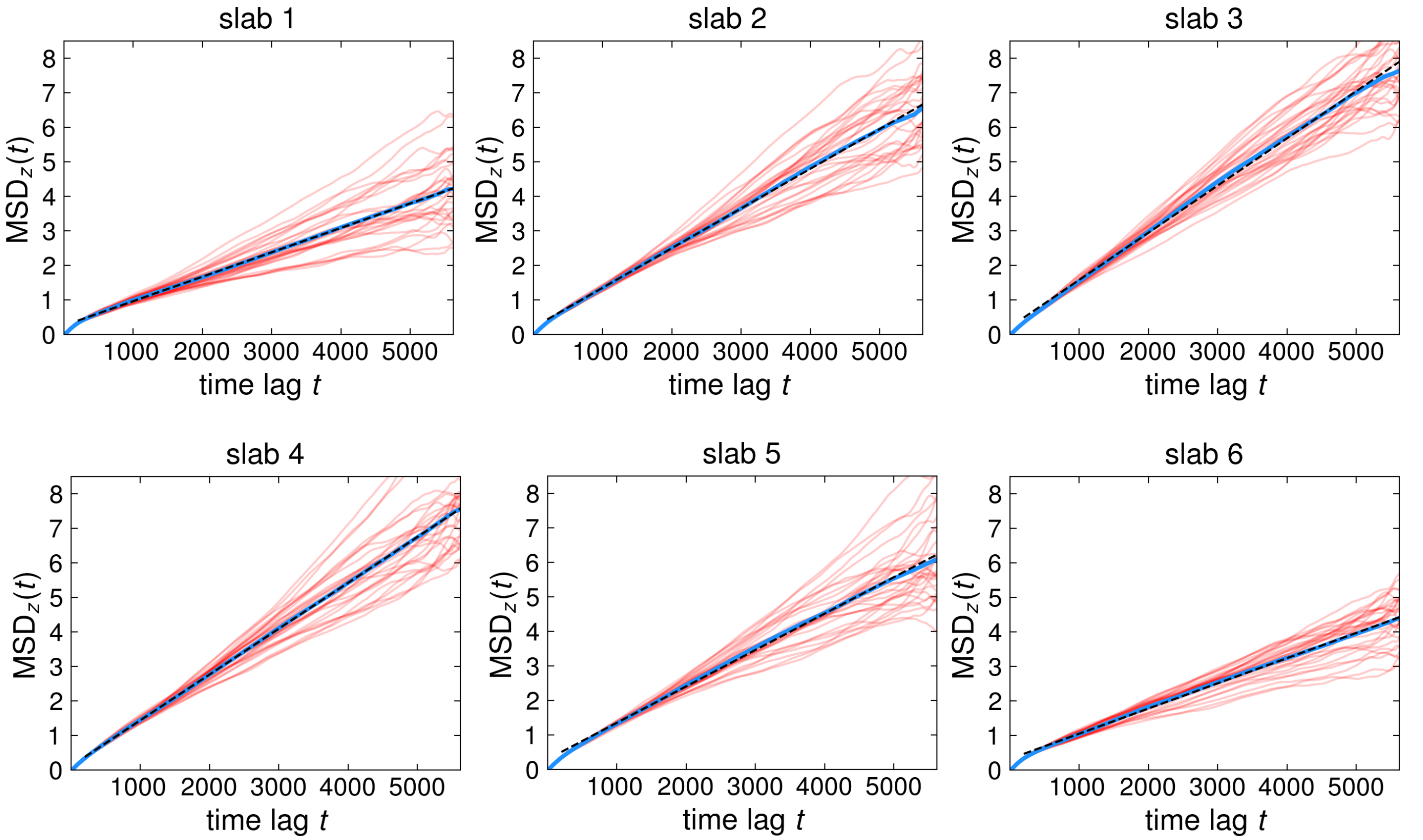

Figure S15: Average MSD along z direction (MSD_z) of confined l-GST-225 in each slab as a function of the timestep at $T = 1300$ K. Red curves relate to MSD_z of different sub-runs of the AIMD trajectory. The blue curve is the Average MSD_z and the dashed black line is a linear fit of the MSD_z in the diffusion regime.

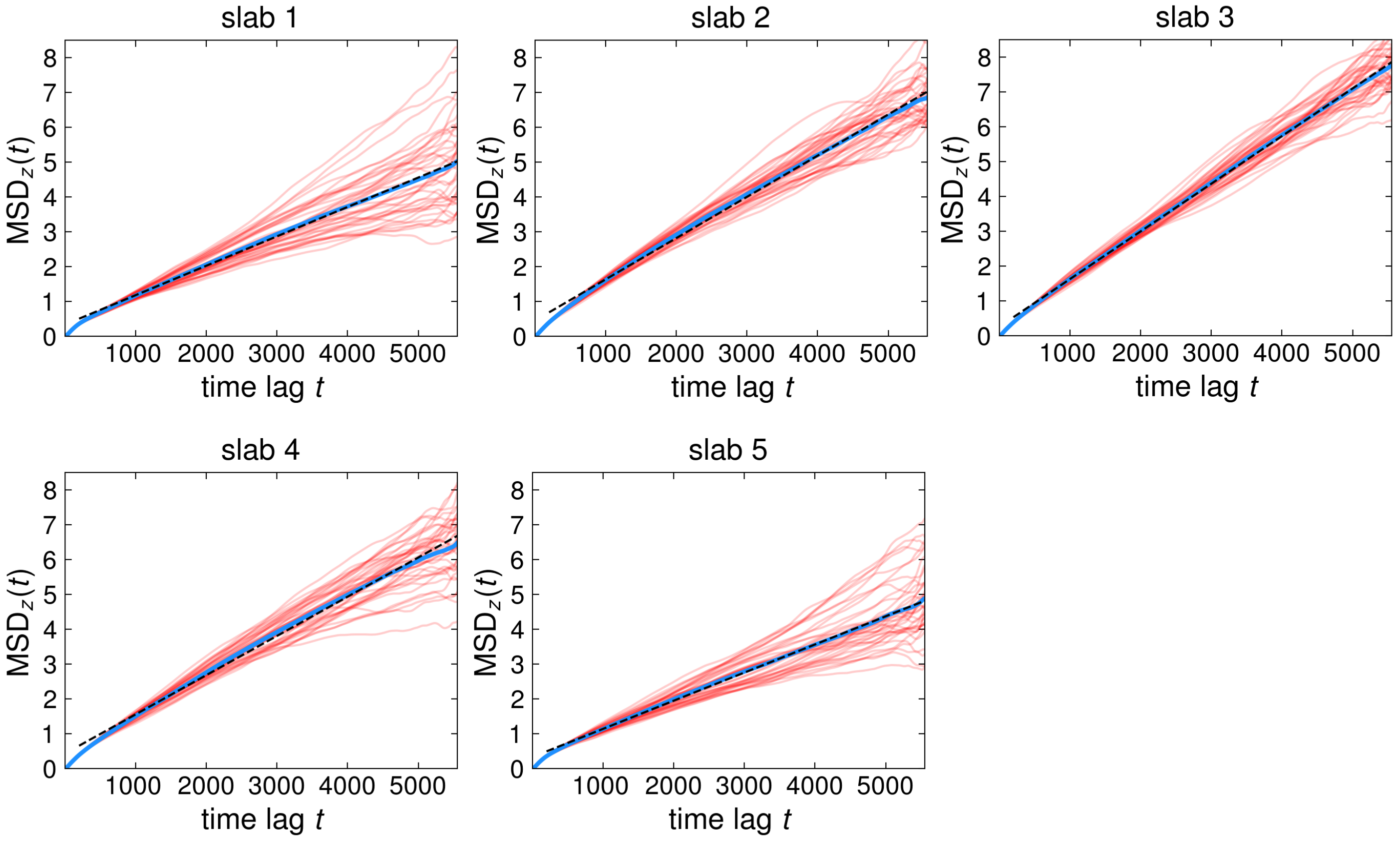

Figure S16: Average MSD along z direction (MSD_z) of confined l-GeTe in each slab as a function of the timestep at $T = 1300$ K. Red curves relate to MSD of different sub-runs of the AIMD trajectory. The blue curve is the Average MSD_z and the dashed black line is a linear fit of the MSD_z in the diffusion regime.

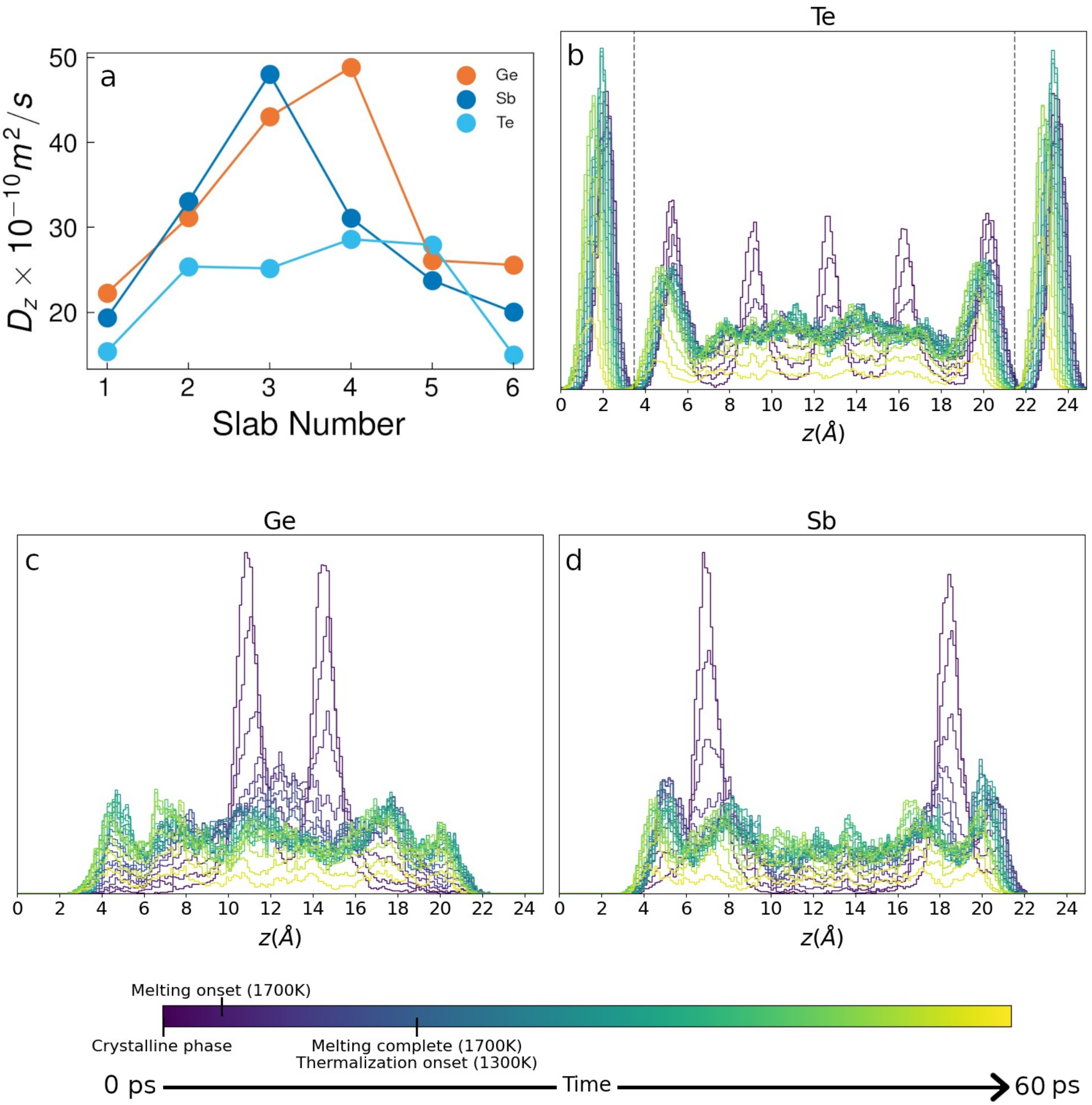

Figure S17: Inset a) D_z self-diffusion coefficient profiles in confined GST-225 at T = 1300 K. Ge and Sb atoms exhibit comparable trends, whereas the mobility of Te atoms is generally suppressed. Insets b-d) show various Te, Ge and Sb density profiles in the PCH. Each curves is generated by sampling the MD trajectory at different times: starting from a pre-melted crystalline phase (purple curves), passing through an overheated phase (Melting onset - melting complete on the colorbar) and finally reaching a thermalized liquid phase at 1300 K (Thermalization onset on the colorbar). These curves indicate that the decrease in mobility of the Te atoms is due to a layering effect induced in the PCM by the interface. Indeed, the two peaks corresponding to the Te layers of GST-225 next to the $TiTe_2$ trilayer disappear slowly (contrary to the peaks corresponding to the Ge and Sb layers) and non-negligible peaks are present even in the fully liquid state. This is in line with the fact that the presence of the Te layers of the confinement material favors the presence of Te-rich regions near the interfaces, resulting in lower mobility for this atomic species.

6 Amorphous phase

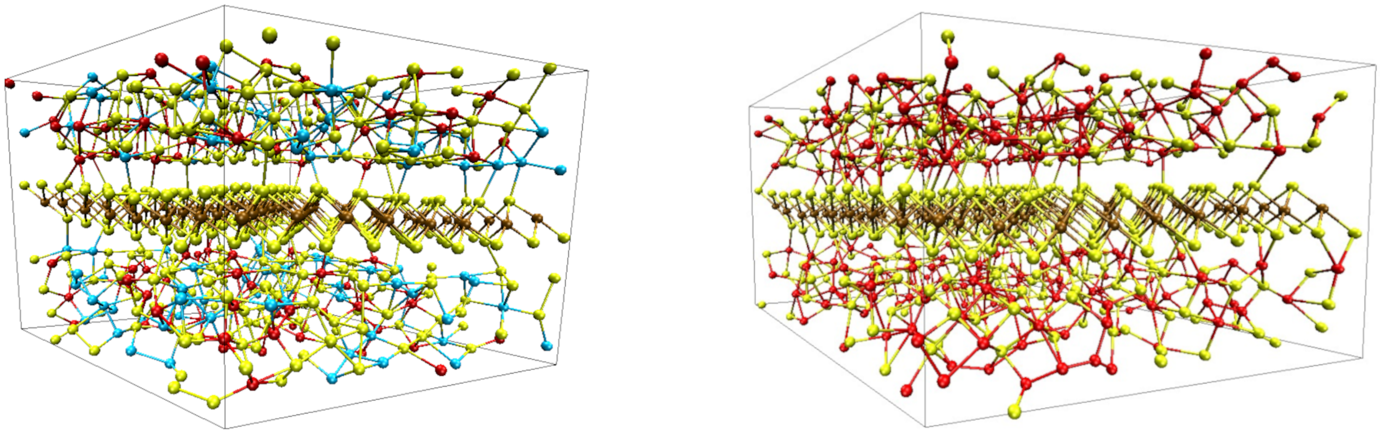

Figure S18: Snapshots of a-GST-225 (left) and a-GeTe (right) at $T = 300$ K. We depict Te, Ti, Ge and Sb atoms in yellow, brown, red and cyan respectively.

6.1 Coordination

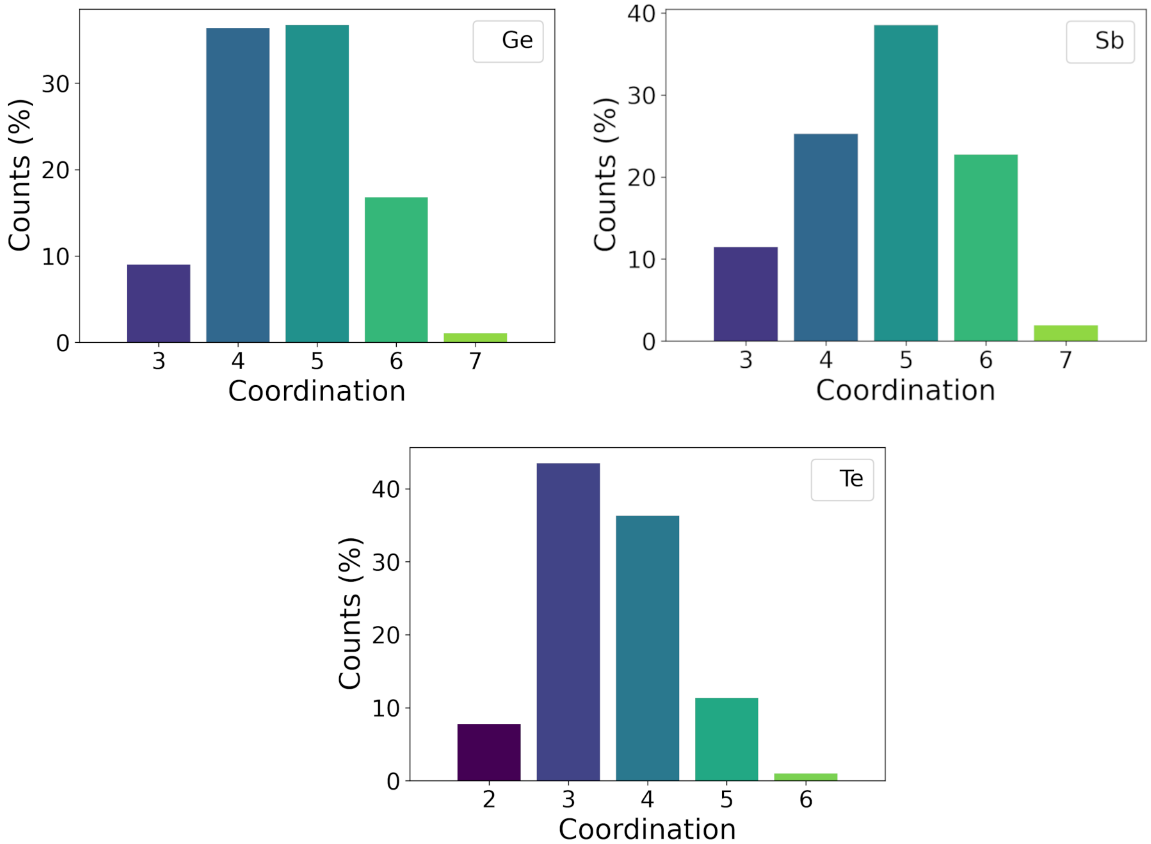

Figure S19: Coordination of a-GST-225 at $T = 300$ K. The upper plot shows Ge (left) and Sb (right) coordination; The lower plot shows Te coordination.

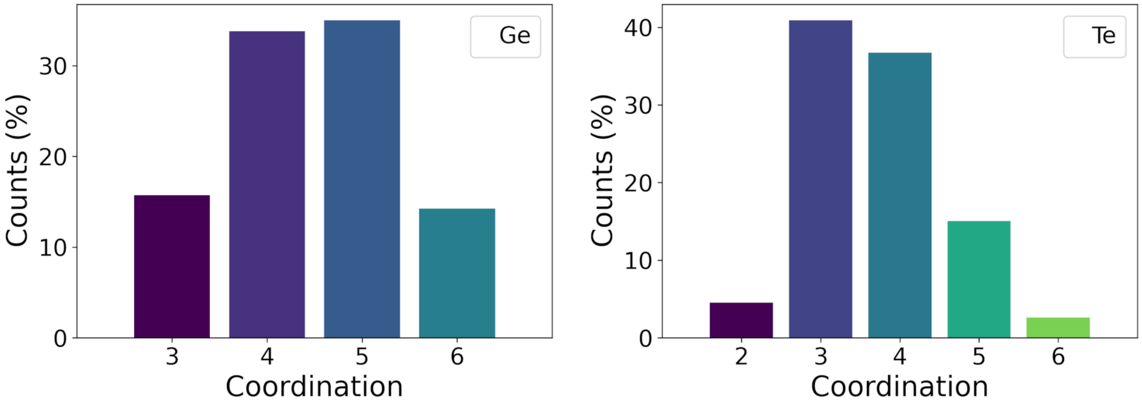

Figure S20: Coordination of a-GeTe at $T = 300$ K. The left plot shows Ge coordination; the right plot shows Te coordination

6.2 Coordination Numbers

The total and partial CN of Refs.[9, 10], can be found by setting $r_{cut} = 3.2$ Å for all $\alpha\beta$ pairs with the exception of the Sb-Te pair where $r_{cut} = 3.4$ Å. These values are included in both scatter plots of **Figures(S21)** and **(S22)** but we also report a comparison of our values with theirs in **Table S5**. We observe a slight increase of N_{TeTe} with respect to [9, 10].

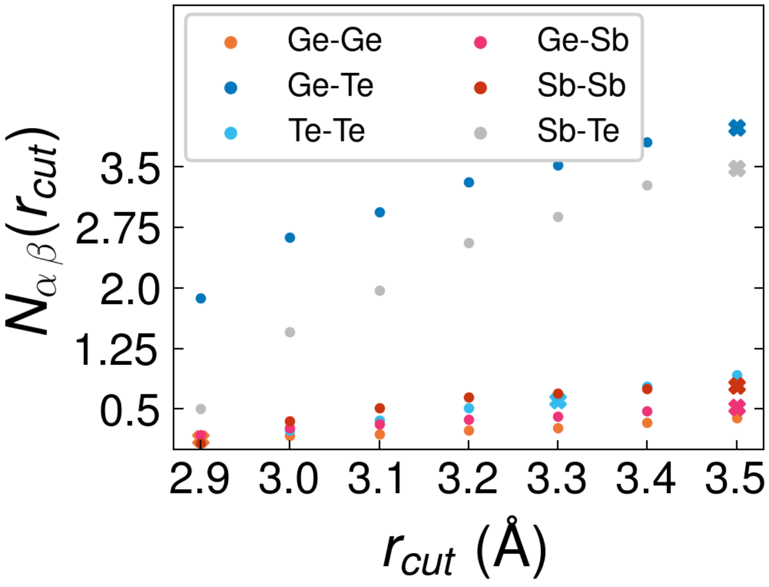

Figure S21: CN $N_{\alpha\beta}$ of confined a-GST-225 as a function of r_{cut} at $T = 300$ K. Different colors refer to different $\alpha\beta$ atomic pairs. Crosses represent the CN values that we obtained by means of the cutoff radii specified in Table 9 of the main text.

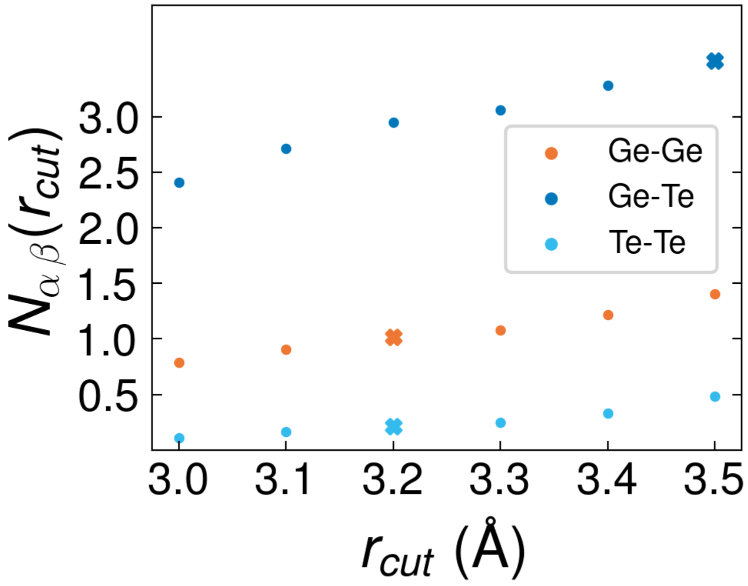

Figure S22: CN $N_{\alpha\beta}$ of confined a-GeTe as a function of r_{cut} at $T = 300$ K. Different colors refer to different $\alpha\beta$ atomic pairs. Crosses represent the CN values that we obtained by means of the cutoff radii specified in Table 9 of the main text.

Pair	r_{cut} (Å) Ref. [9, 10]	$N_{\alpha\beta}$ Ref. [9, 10]	$N_{\alpha\beta}$
Ge-Ge	3.2	0.275	0.234
Ge-Sb	3.2	0.270	0.370
Ge-Te	3.2	3.227	3.310
Sb-Sb	3.2	0.588	0.643
Sb-Te	3.4	3.166	3.270
Te-Te	3.2	0.288	0.512

Table S5: Comparison of CNs concerning a-GST-225. Column 2 outlines the cutoff radii utilized in the works of Caravati et al. [9, 10], while column 3 presents the overall CNs reported by the same authors in their work. In column 4, we present the total CNs we obtained when applying the cutoffs specified in column 2.

6.3 Sub and Partial Angular Distribution Function

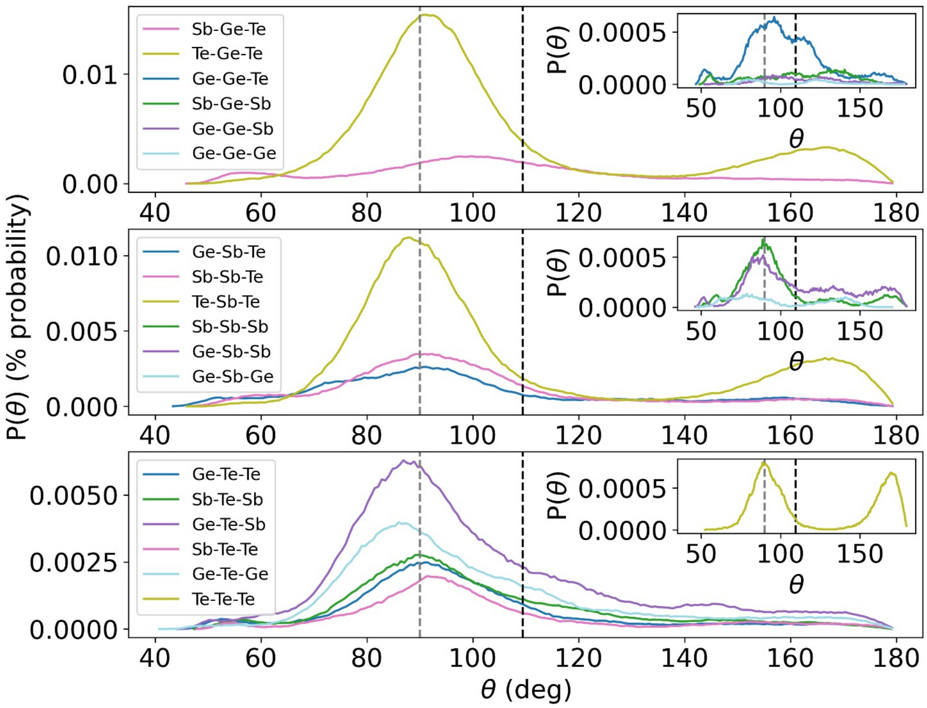

Figure S23: Partial ADFs (pADFs) of a-GST-225 at $T = 300$ K. The upper plot shows all the Ge-centered triplets; the mid plot shows all the Sb-centered triplets; the lower plot shows all the Te-centered triplets. Each pADF is normalized to the relative number of atomic triplets. The grey dashed line is centered at 90° while the black dashed line is centered at 109.5°.

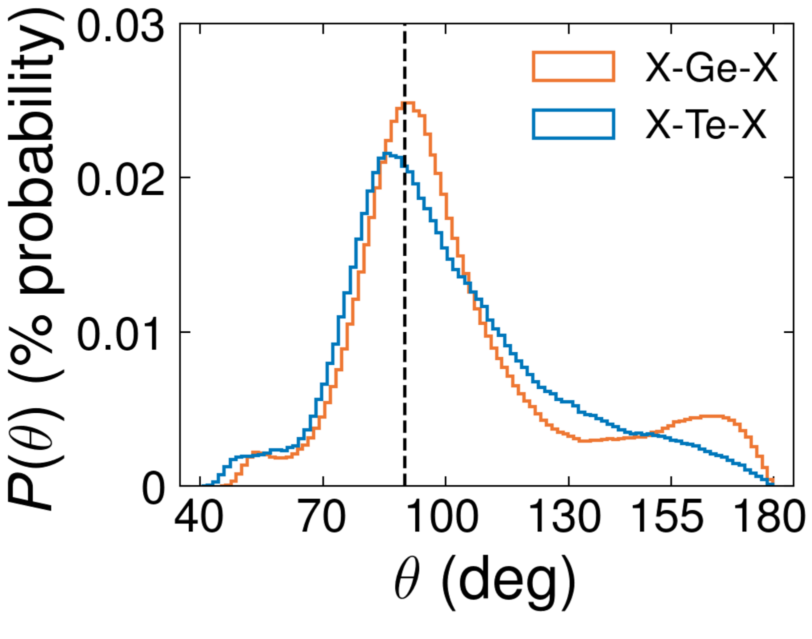

Figure S24: sub ADFs of a-GeTe at $T = 300$ K. Each sub-ADF is normalized to the relative number of atomic triplets. The black dashed line is centered at 90°.

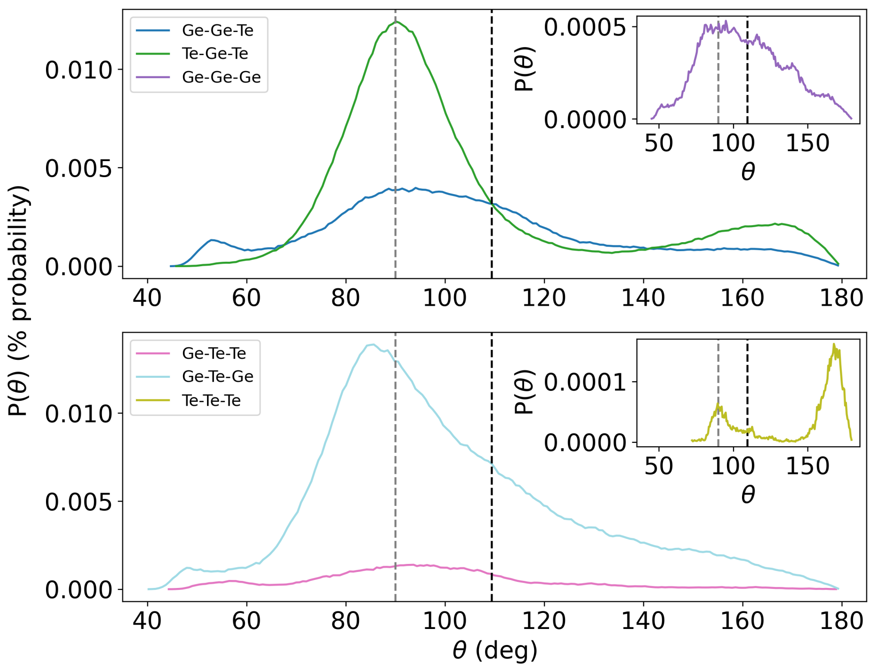

Figure S25: Partial ADFs (pADFs) of a-GeTe. The upper plot shows all the Ge-centered triplets; the lower plot shows all the Te-centered pADFs. Each pADF is normalized to the relative number of atomic triplets. The grey dashed line is centered at 90° while the black dashed line is centered at 109.5°.

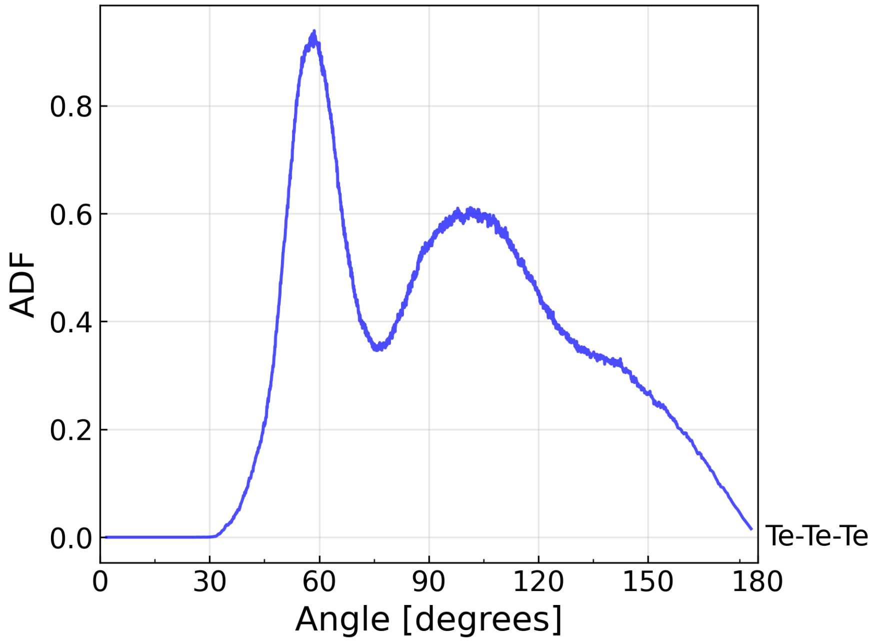

Figure S26: Te-Te-Te ADF of confined a-GeTe at $T = 300$ K upon inclusion of the second Te-Te coordination shell at $r_{cut} \approx 5.22$ Å. We observe some discrepancies between our ADF and the one of Ref. [40] in the main text: in particular, we do not observe clear features at 90° and 120° degrees, contrary to their models. These discrepancies appear to be due to confinement effects. Calculation of the Te-Te-Te ADF for an independent a-GeTe bulk model at $T = 300$ K obtained with a machine-learning potential leads to more satisfactory agreement with the findings of Ref. [40], as the two features at 90° and 120° degrees are better resolved.

6.4 q order parameter

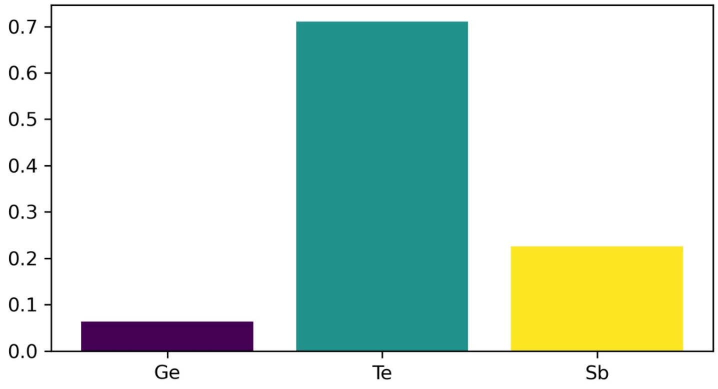

Figure S27: Nearest neighbors of the Ge atoms with tetrahedral coordination, 6.3% are Ge, 71% are Te, and 22.6% are Sb.

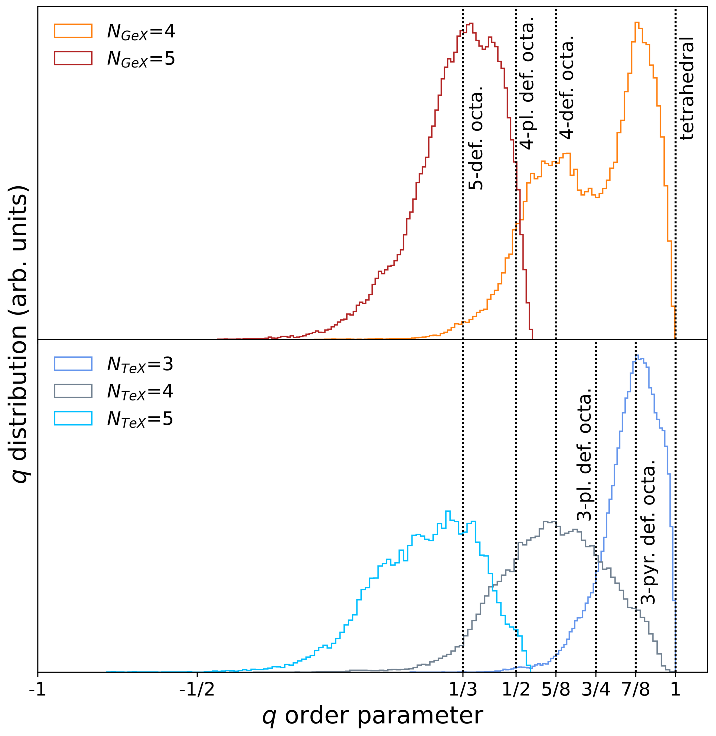

Figure S28: Errington-De Benedetti local order parameter q of a-GeTe at T = 300 K. The upper plot shows the 4 and 5-fold coordination of Ge atoms; the lower plot shows the 3, 4, and 5-fold coordination of Te atoms.

6.5 Distribution of Primitive Rings

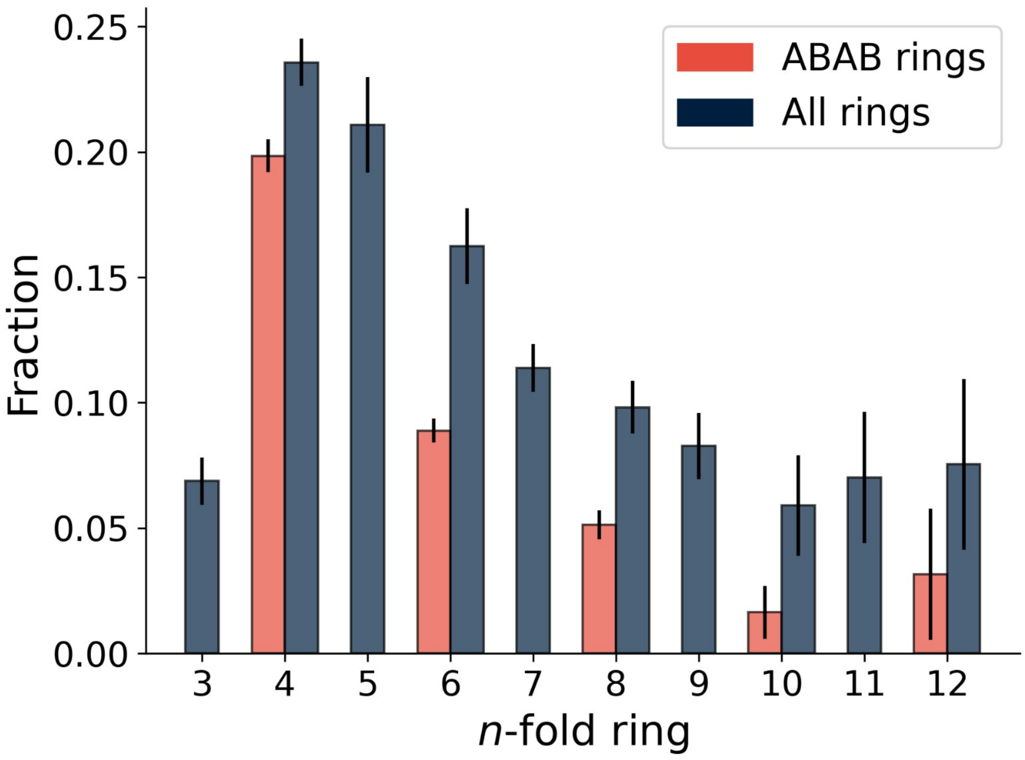

Figure S29: Distribution of primitive rings in a-GeTe at $T = 300$ K. The vertical black bars in the ring histogram are error bars computed with the RINGS code.

References

[1] Thomas D Kühne et al. "CP2K: An electronic structure and molecular dynamics software package-Quickstep: Efficient and accurate electronic structure calculations". In: *The Journal of Chemical Physics* 152.19 (2020).

[2] S. Goedecker, M. Teter, and J. Hutter. "Separable dual-space Gaussian pseudopotentials". In: *Physical Review B* 54.3 (July 1996), pp. 1703–1710. ISSN: 1095-3795. DOI: 10.1103/physrevb.54.1703. URL: http://dx.doi.org/10.1103/PhysRevB.54.1703.

[3] Stefan Grimme et al. "A consistent and accurate ab initio parametrization of density functional dispersion correction (DFT-D) for the 94 elements H-Pu". In: *The Journal of chemical physics* 132.15 (2010).

[4] Stefan Grimme. "Semiempirical GGA-type density functional constructed with a long-range dispersion correction". In: *Journal of Computational Chemistry* 27.15 (Sept. 2006), pp. 1787–1799. ISSN: 1096-987X. DOI: 10.1002/jcc.20495. URL: http://dx.doi.org/10.1002/jcc.20495.

[5] Thomas D Kühne et al. "Efficient and accurate Car-Parrinello-like approach to Born-Oppenheimer molecular dynamics". In: *Physical review letters* 98.6 (2007), p. 066401.

[6] Giovanni Bussi and Michele Parrinello. "Accurate sampling using Langevin dynamics". In: *Phys. Rev. E* 75 (5 2007), p. 056707. DOI: 10.1103/PhysRevE.75.056707. URL: https://link.aps.org/doi/10.1103/PhysRevE.75.056707.

[7] Hans Weber et al. "Experimental and ab initio molecular dynamics study of the structure and physical properties of liquid GeTe". In: *Physical Review B* 96.5 (2017), p. 054204.

[8] Mathias Schumacher et al. "Structural, electronic and kinetic properties of the phase-change material Ge2Sb2Te5 in the liquid state". In: *Scientific reports* 6.1 (2016), p. 27434.

[9] S Caravati et al. "First-principles study of crystalline and amorphous Ge2Sb2Te5and the effects of stoichiometric defects". In: *Journal of Physics: Condensed Matter* 21.25 (2009), p. 255501. ISSN: 1361-648X. DOI: 10.1088/0953-8984/21/25/255501. URL: http://dx.doi.org/10.1088/0953-8984/21/25/255501.

[10] S Caravati et al. “Coexistence of tetrahedral-and octahedral-like sites in amorphous phase change materials”. In: *Applied Physics Letters* 91.17 (2007).
